# Cell-free chromatin from dying cancer cells integrate into genomes of bystander healthy cells to induce DNA damage and inflammation

**DOI:** 10.1038/cddiscovery.2017.15

**Published:** 2017-05-29

**Authors:** Indraneel Mittra, Urmila Samant, Suvarna Sharma, Gorantla V Raghuram, Tannistha Saha, Pritishkumar Tidke, Namrata Pancholi, Deepika Gupta, Preeti Prasannan, Ashwini Gaikwad, Nilesh Gardi, Rohan Chaubal, Pawan Upadhyay, Kavita Pal, Bhagyeshri Rane, Alfina Shaikh, Sameer Salunkhe, Shilpee Dutt, Pradyumna K Mishra, Naveen K Khare, Naveen K Nair, Amit Dutt

**Affiliations:** 1 Translational Research Laboratory, Advanced Centre for Treatment, Research and Education in Cancer, Tata Memorial Centre, Mumbai 410210, India; 2 Division of Laboratory Medicine, Tata Memorial Hospital, Tata Memorial Centre, Mumbai 400012, India; 3 Integrated Cancer Genomics Laboratory, Advanced Centre for Treatment, Research and Education in Cancer, Tata Memorial Centre, Mumbai 410210, India; 4 Homi Bhabha National Institute, Training School Complex, Anushakti Nagar, Mumbai 410210, India; 5 DNA Repair and Chromatin Biology Laboratory, Advanced Centre for Treatment, Research and Education in Cancer, Tata Memorial Centre, Mumbai 410210, India

## Abstract

Bystander cells of the tumor microenvironment show evidence of DNA damage and inflammation that can lead to their oncogenic transformation. Mediator(s) of cell–cell communication that brings about these pro-oncogenic pathologies has not been identified. We show here that cell-free chromatin (cfCh) released from dying cancer cells are the key mediators that trigger both DNA damage and inflammation in the surrounding healthy cells. When dying human cancer cells were cultured along with NIH3T3 mouse fibroblast cells, numerous cfCh emerged from them and rapidly entered into nuclei of bystander NIH3T3 cells to integrate into their genomes. This led to activation of H2AX and inflammatory cytokines NF*κ*B, IL-6, TNF*α* and IFN*γ*. Genomic integration of cfCh triggered global deregulation of transcription and upregulation of pathways related to phagocytosis, DNA damage and inflammation. None of these activities were observed when living cancer cells were co-cultivated with NIH3T3 cells. However, upon intravenous injection into mice, both dead and live cells were found to be active. Living cancer cells are known to undergo extensive cell death when injected intravenously, and we observed that cfCh emerging from both types of cells integrated into genomes of cells of distant organs and induced DNA damage and inflammation. *γ*H2AX and NF*κ*B were frequently co-expressed in the same cells suggesting that DNA damage and inflammation are closely linked pathologies. As concurrent DNA damage and inflammation is a potent stimulus for oncogenic transformation, our results suggest that cfCh from dying cancer cells can transform cells of the microenvironment both locally and in distant organs providing a novel mechanism of tumor invasion and metastasis. The afore-described pro-oncogenic pathologies could be abrogated by concurrent treatment with chromatin neutralizing/degrading agents suggesting therapeutic possibilities.

## Introduction

Bystander effect is a phenomenon typically associated with ionizing radiation wherein non-targeted cells show evidence of heritable DNA damage and genomic instability.^1^ These effects are thought to result from damaging substances released from irradiated cells, as conditioned media of treated cells can induce damage in non-irradiated recipient cells.^2^ Cells of distant tissues also exhibit bystander or abscopal effects, which can have oncogenic consequences.^3,4^ Radio-sensitive mice have been reported to develop cancerous changes in the cerebellum following X-ray exposure to the body after the cranium is carefully shielded.^5^ Bystander effect is being increasingly recognized in relation to cancer. Non-cancerous cells of the tumor microenvironment show evidence of DNA damage response (DDR) in the form of positive staining for *γ*-H2AX and phospho-ATM.^6^ Bystander effect is not restricted to peri-tumoural cells alone, tumor-related DNA damage in cells of distant tissues have been reported *in vivo*.^7,8^ Mice bearing sub-cutaneous tumors show signs of DNA damage in several tissues, being most pronounced in proliferating cells.^7^ Inflammation is another long recognized component of the oncogenic process and an inflammatory microenvironment is evident in most cancers.^9,10^ The key features of the cancer-related inflammatory microenvironment include infiltration by lymphocytes and macrophages, presence of inflammatory cytokines and vascular endothelial growth factors involved in neo-vascularization and angiogenesis.^11–13^ Cancer is also associated with systemic inflammation similar to that observed in infections or inflammatory diseases.^14–16^ Cancer patients exhibit elevated serum levels of inflammatory cytokines and acute-phase proteins and alteration in peripheral blood count, gene expression patterns and erythrocyte sedimentation rates.^14,17,18^

Identification of agents responsible for inter-cellular communication that induce local and systemic bystander DNA damage and inflammation have remained elusive. We have recently reported that circulating cell-free chromatin (cfCh) isolated from serum of cancer patients can readily enter into nuclei of healthy cells to integrate into their genomes triggering DDR, dsDNA breaks and apoptosis both *in vitro* and *in vivo*.^19–21^ We hypothesized that cfCh that are released from dying cancer cells might similarly enter into bystander healthy cells of the tumor microenvironment and those of distant organs, activate DDR and damage their DNA. As dying cells are known to induce an inflammatory response in surrounding cells,^22,23^ we further hypothesized that cfCh from dying cancer cells might also be responsible for tumor-related inflammation in surrounding bystander cells. We show here that when dying cancer cells were co-cultivated with NIH3T3 cells or injected into mice, cfCh emerging from them readily entered into bystander cells and integrated into their genomes as detected by PCR and fluorescence *in situ* hybridization (FISH). Genomic integration induced dsDNA breaks, global transcriptional deregulation and intense upregulation of inflammatory cytokines. There is a large body of literature to show that DNA damage and inflammation act synergistically to drive the oncogenic process.^11,24^ Our results suggest that cfCh are the key agents that bring about both these inter-related pathologies in bystander cells of the tumor microenvironment and in those of distant tissues to create a pro-oncogenic milieu that could promote their cancerous transformation.

## Results

### Cellular uptake and nuclear accumulation of cfCh *in vitro*

**Figure 1:**
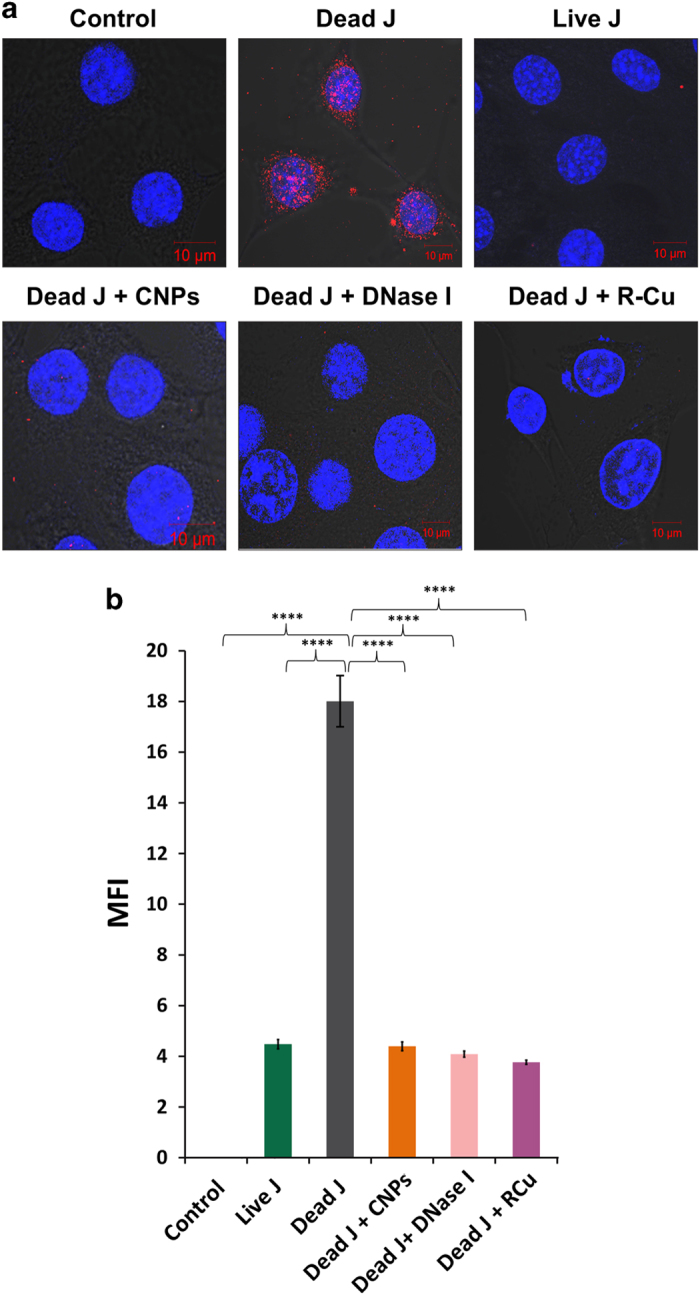
Cellular uptake and nuclear accumulation of cfCh and their prevention by chromatin neutralizing/degrading agents. (**a**) Representative laser scanning confocal microscopy images showing nuclear uptake of numerous fluorescent particles by NIH3T3 cells released from dead Jurkat cells pre-labeled with BrdU at 6 h. The uptake of cfCh is prevented by concurrent treatment with CNPs, DNase I and R-Cu. Few nuclear fluorescent signals are seen when live Jurkat cells were used. (**b**) Quantitative analysis of mean fluorescence intensity (MFI) of images given in **a**. Fifty nuclei were analyzed for quantifying MFI. Data were analyzed by Student’s *t*-test. ****P=0.0001. J, Jurkat cells.

BrdU-labeled dead Jurkat cells were co-cultivated with NIH3T3 cells and examined by confocal microscopy at 6 h. Numerous fluorescently labeled particles could be seen within the NIH3T3 cells, particularly within their nuclei (Figures 1a and b). Few intra-nuclear fluorescent particles were detectable when live Jurkat cells were used (Figures 1a and b). Nuclear uptake of fluorescent particles from dead Jurkat cells could be markedly inhibited (*P*<0.0001) by concurrent treatment with three chromatin neutralizing/degrading agents namely, anti-histone antibody complexed nanoparticles (CNPs), DNase I and resveratrol-copper (R-Cu) (Figures 1a and b). This finding that BrdU particles could be equally effectively eliminated by both anti-histone antibody CNPs as well as DNA-degrading agents indicated that BrdU particles were nothing other than chromatin fragments that contained both histones and DNA. Negative control experiments using the metabolic poison actinomycin D and those conducted at low temperature (31 °C) indicated that the uptake of cfCh from dead Jurkat cells was cellular energy dependent; there being significant reduction (*P*<0.0001) in uptake of fluorescently labeled cfCh under both these conditions (Supplementary Figure 1). A time-course analysis of uptake of fluorescent cfCh from dead Jurkat cells by nuclei of recipient NIH3T3 cells showed that nearly 100% of the cells had taken up cfCh by 3 h remaining elevated for the next 48 h followed by a sharp decline (Supplementary Figure 2).

### Induction of bystander DDR and inflammation *in vitro*

**Figure 2:**
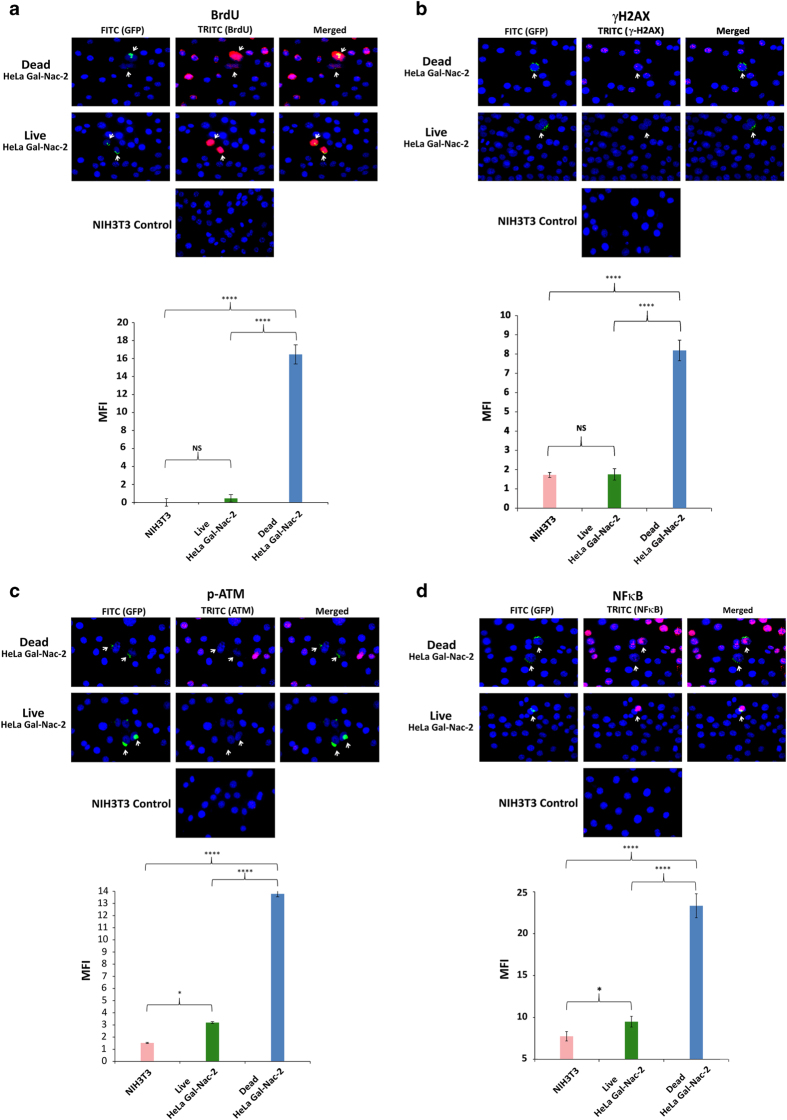

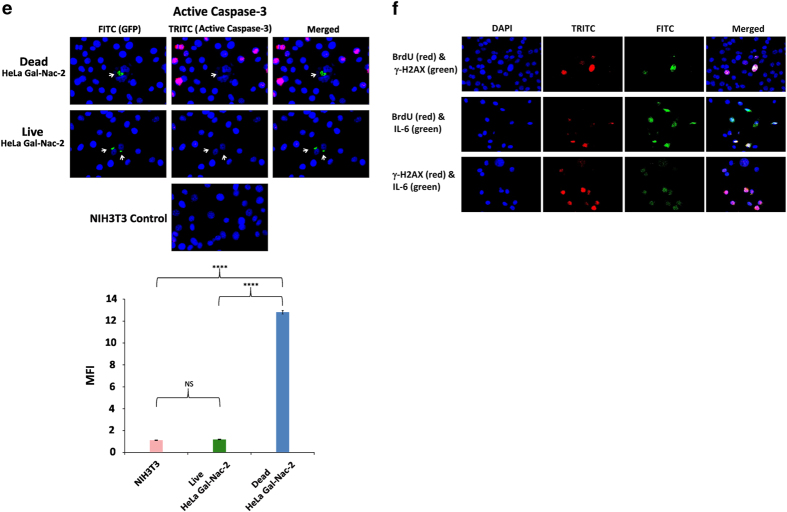
Bystander uptake of fluorescently labeled cfCh and activation of DNA damage and inflammation following co-cultivation of NIH3T3 cells with irradiated and un-irradiated GalNAc-T2-GFP HeLa cells. (**a**) Upper panel, fluorescent microscopy at 36 h showing uptake by bystander NIH3T3 cells of fluorescent cfCh particles released from BrdU pre-labeled dead, but not live, GalNAc-T2-GFP HeLa cells; lower panel, quantitative analysis of mean fluorescence intensity (MFI) of images given in upper panel. (**b**–**e**) Upper panels, fluorescent microscopy at 36 h showing activation of H2AX (**b**), p-ATM (**c**), NF*κ*B (**d**) and active Caspase-3 (**e**) in bystander NIH3T3 cells. Lower panels, quantitative analysis of MFI of images given in upper panel. One thousand nuclei were gated and analyzed for quantifying MFI in each case. *P = 0.05; ****P = 0.0001; NS=Not Significant. Results (mean±S.E.) were analyzed by Student’s *t*-test. (**f**) Fluorescent microscopy of NIH3T3 cells co-cultivated with irradiated B16-F10 melanoma cells at 6 h showing BrdU and *γ*-H2AX co-expressing cells (upper panel); BrdU and IL-6 co-expressing cells (middle panel), and *γ*-H2AX and IL-6 co-expressing cells (lower panel).

As it was our original hypothesis that cfCh might be responsible for bystander DNA damage and inflammation, we conducted experiments in which irradiated BrdU-labeled dying cells were co-cultivated with NIH3T3 cells at a ratio of 1 : 20. We used BrdU-labeled GalNAc-T2-GFP HeLa cells and irradiated them so that the cfCh-donor dead cells could be identified by virtue of their GFP green fluorescence. We clearly observed that BrdU-labeled cfCh particles that had emerged from irradiated GalNAc-T2-GFP HeLa cells had entered into nuclei of bystander living NIH3T3 cells (Figure 2a, upper panel). BrdU particles were not seen in the bystander cells when un-irradiated GalNAc-T2-GFP HeLa cells were used (Figure 2a, upper panel). Quantitative estimation of BrdU mean fluorescence intensity of control, irradiated and un-irradiated donor GalNAc-T2-GFP HeLa cells is given in Figure 2a lower panel, which clearly shows that BrdU particles are released from irradiated but not from un-irradiated cells. We also detected activation of H2AX and p-ATM in the bystander cells indicative of dsDNA breaks and DDR activation (Figures 2b and c). The latter were not seen when un-irradiated GalNAc-T2-GFP HeLa cells were co-cultured with NIH3T3 cells (Figures 2b and c). We further confirmed the activation of H2AX and p-ATM by western blot analysis. Supplementary Figure 3 clearly shows the presence of the expected *γ*H2AX and p-ATM bands in case of irradiated GalNAc-T2-GFP HeLa cells but not in the case of un-irradiated HeLa cells. Bystander NIH3T3 cells also showed evidence of inflammation, which was confirmed by our detection of the pro-inflammatory transcription factor NF*κ*B (Figure 2d). Evidence of apoptosis as indicated by active Caspase-3 expression in the bystander cells was also evident in the presence of irradiated GalNAc-T2-GFP HeLa cells but was absent when un-irradiated HeLa cells were used (Figure 2e).

Evidence that cfCh were directly responsible for DNA damage and inflammation in bystander cells is provided in Figure 2f in which NIH3T3 cells were co-cultivated with irradiated B16-F10 melanoma cells. We clearly observed co-localization of BrdU-labeled fluorescent cfCh signals with those of *γ*H2AX (Figure 2f, upper panel) and IL-6 (Figure 2f, middle panel) confirming that cfCh were directly responsible for H2AX and IL-6 activation. Significantly, we also observed that *γ*H2AX and IL-6 were often co-expressed by the same bystander cells (Figure 2f, lower panel) indicating that DNA damage and inflammation are closely linked pathologies that are concurrently activated. Further evidence of co-activation of DNA damage and inflammation is provided in Supplementary Figure 4 in which NIH3T3 cells were co-cultured with irradiated dying GalNAc-T2-GFP HeLa cells. It clearly shows co-localization of several fluorescent signals of *γ*H2AX and NF*κ*B as indicated by arrows.

### Induction of bystander DDR and inflammation *in vivo*

**Figure 3:**
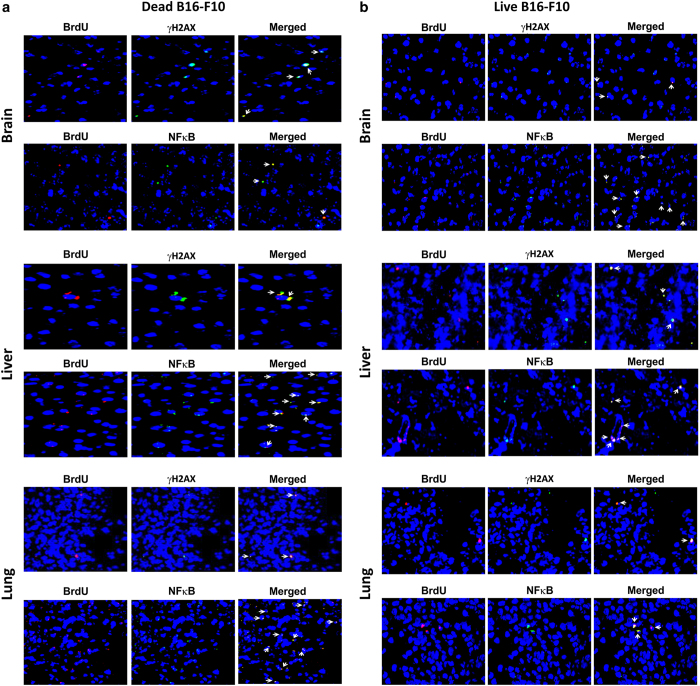
Detection of fluorescent signals in nuclei of cells of vital organs of mice following i.v. injection of dead and live B16-F10 cells pre-labeled with BrdU and activation of H2AX and NF*κ*B. One hundred thousand BrdU pre-labeled dead and live B16-F10 cells were injected i.v. into mice and animals were killed after 72 h. (**a**, **b**) Detection of fluorescent BrdU signals (left hand panels) and activation of H2AX and NF*κ*B (middle panels) in brain, liver and lung following i.v. injection of dead and live B16-F10 cells. It is noteworthy that BrdU signals frequently co-localize with fluorescent signals generated by H2AX and NF*κ*B activation (right hand panels).

Upon intravenous injection into mice, dying cancer cells that reached distant organs could release cfCh and induce bystander DDR and inflammation in their cells. It is well-established that living cancer cells when injected intravenously undergo extensive cell death as a consequence of the shearing force of circulation.^25,26^ We used B16-F10 melanoma cells, rather than Jurkat cells, and injected them into isogenic C57/Bl6 mice. Jurkat cells were not used because it could be argued that Jurkat cells (human) might die when injected into mice, not because of shearing forces of circulation (and other factors), but because of an immune reaction mounted by the host due to cross-species incompatibility. As living B16-F10 melanoma cells are known to undergo extensive cell death upon intravenous injection,^25^ our hypothesis was that both dying and living B16-F10 cells would release cfCh particles into cells of the target organs upon reaching their respective destinations. Indeed, BrdU signals could be clearly detected in brain, lung and liver cell nuclei of injected mice indicating that cfCh from both dead and living B16-F10 melanoma cells had entered bystander cells of distant organs (Figures 3a and b, left hand panels). We also demonstrate activation of H2AX and NF*κ*B in all three organs in case of both dying and living cells indicating activation of bystander DNA damage and inflammation (Figures 3a and b, middle panels). Significantly, BrdU fluorescent signals and those of *γ*H2AX and NF*κ*B often co-localized indicating that cfCh were directly responsible for activation of DNA damage and inflammation (Figures 3a and b, right hand panels). DAPI images of these organs from un-injected mice were given in Supplementary Figure 5.

**Figure 4:**
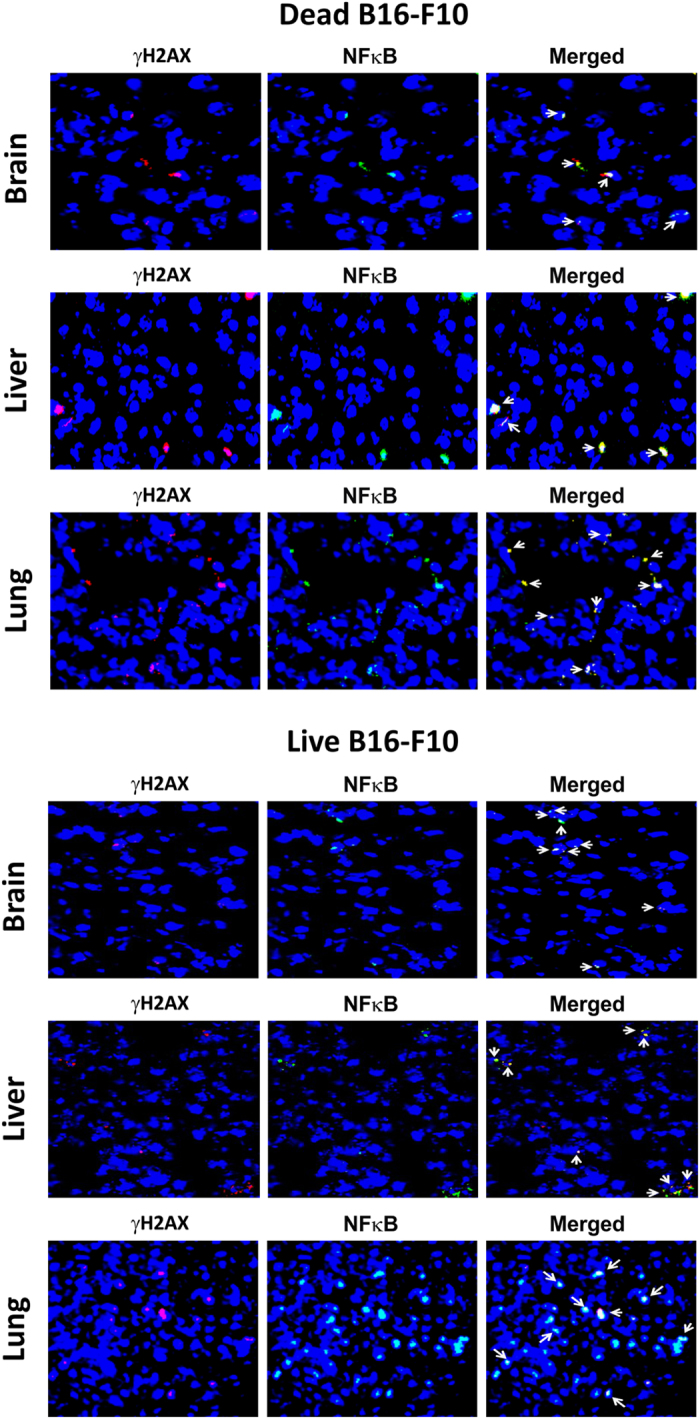
Co-localization of *γ*H2AX and NF*κ*B fluorescent signals in vital organs of mice following intravenous injection of dead (upper panels) and live (lower panels) B16-F10 cells. One hundred thousand dead and live B16-F10 cells were injected i.v. into mice and animals were killed after 72 h.

Finally, we observed, with respect to both dying (Figure 4, upper panel) and living cells (Figure 4, lower panel), that *γ*H2AX and NF*κ*B were co-expressed in the same cells of brain, liver and lung of mice. This is consistent with our *in vitro* findings that DNA damage and inflammation are closely linked pathologies and are activated through yet unidentified common pathway(s).

We undertook further control experiments in which we injected free BrdU alone to investigate whether BrdU itself could be responsible for the above findings. We arbitrarily injected into mice the same concentration of BrdU that we normally use for labeling cultured cells *in vitro* (13 *μ*M) equated for blood volume of mice (5 ml). This dose is likely to be several orders of magnitude higher than the BrdU label contained in cfCh particles when dead or live B16-F10 melanoma cells were injected. Therefore, we also injected in the same experiments BrdU-labeled live and dead B16-F10 melanoma cells as comparators (Supplementary Figure 6A). The number of fluorescent signals following free BrdU injection was significantly lower compared to those observed following injection of fluorescently BrdU-labeled dead and live cancer cells indicating that BrdU signals in cells of distant organs were largely due to integration of labeled cfCh particles in their constituent cells. Free BrdU administration also failed to activate H2AX and NF*κ*B in the three vital organs examined namely, brain, liver and lung (Supplementary Figure 6B).

### Detection of transcriptional deregulation by microarray

**Figure 5:**
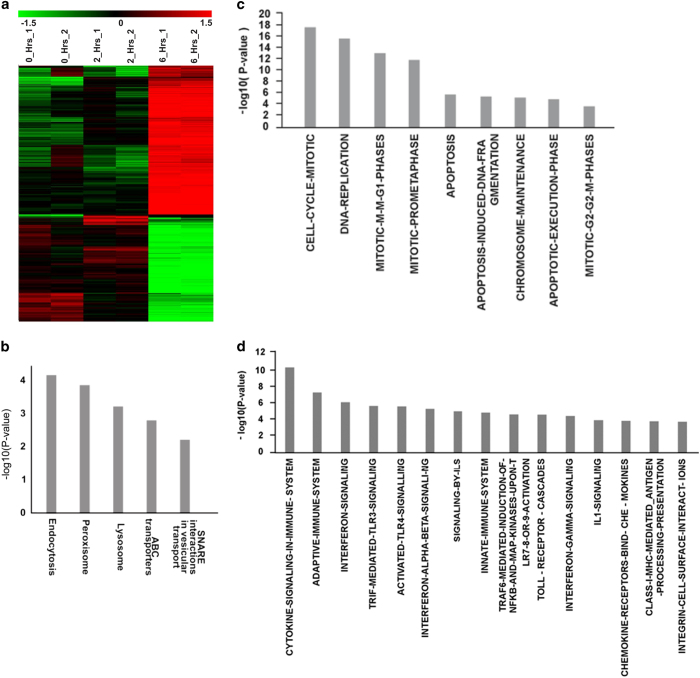
Microarray and pathway analysis of NIH3T3 cells treated with dead Jurkat cells. (**a**) Heat map of significantly differentially expressed genes in NIH3T3 cells treated with dead Jurkat cells at 0, 2 and 6 h in duplicates. Time points are shown in columns and differentially expressed genes in rows. They were grouped together based on the hierarchical clustering method. Red signifies upregulation status, whereas green signifies downregulation. Black color depicts no change in expression. Scale of heat map shown on the top of the figure. (**b**–**d**) Pathway analysis at 6 h showing upregulation of pathways associated with phagocytosis; cell cycle/DNA damage and inflammation, respectively.

We next performed microarray analysis of NIH3T3 cells that had been co-cultivated with dead Jurkat cells at 0, 2 and 6 h. A heat map given in Figure 5a shows that nuclear entry of cfCh had triggered genome-wide transcriptional alterations of NIH3T3 cells after 2 and 6 h. Class comparison between NIH3T3_control (0  h) and NIH3T3+dead Jurkat_2 h resulted in 19 deregulated genes, which suggests that there was minimal transcriptional activation at this time point. However, at 6 h, 817 genes were found to be deregulated when compared to NIH3T3_control (0 h); while a comparison between NIH3T3+dead Jurkat at 2 *versus* 6 h revealed 777 deregulated genes (Figure 5a). When individual class comparison gene lists were combined to make a single gene list, which was representative of genes deregulated in at least one time point, a total of 1004 genes were found to be deregulated (Supplementary Table 2). Analysis of NIH3T3 cells co-cultivated with dead Jurkat cells at 6 h revealed upregulation of several pathways related to phagocytosis; cell cycle/DNA damage and inflammation (Figures 5b–d). The list of genes involved in these pathways is given in Supplementary Table 3.

### Activation of DDR

#### *In vitro* experiments

**Figure 6:**
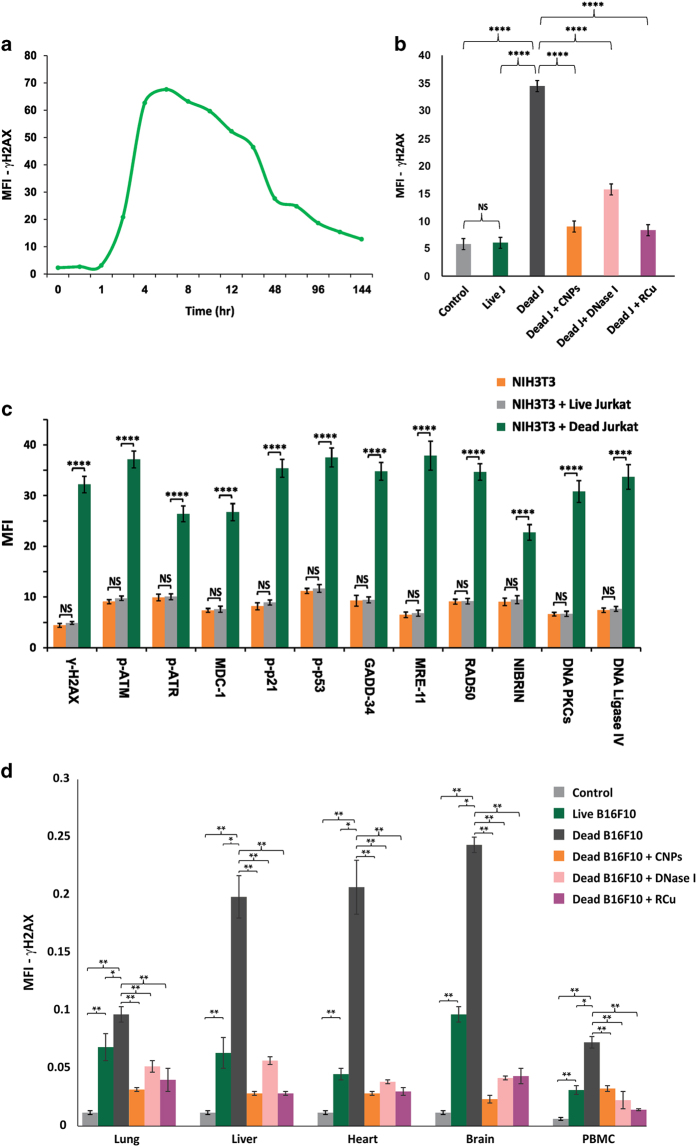
Activation of DDR *in vitro* and *in vivo* and its inhibition by chromatin neutralizing/degrading agents. (**a**) Time course of activation of H2AX in NIH3T3 cells following co-cultivation with dead Jurkat cells as detected by indirect immunofluorescence. The experiment was done in duplicate at each time point and 100 cells were analyzed in each case and the average mean fluorescence intensity (MFI) values are depicted in the graph. (**b**) Prevention of H2AX activation in NIH3T3 cells by CNPs, DNase I and R-Cu following co-cultivation with dead Jurkat cells at 6 h. The experiment was done in duplicate and 500 cells were analyzed in each case for calculating MFI. The histograms depict mean (±S.E.) values in each case. Results were analysed by Student’s *t*-test. ****P<0.0001. Note that live Jurkat cells do not activate H2AX. (**c**) Activation of various DDR and DNA repair proteins following co-cultivation of NIH3T3 cells with dead and live Jurkat cells at 6 h as detected by indirect immunofluorescence. The experiments were done in duplicate and 500 cells were analyzed for calculating MFI. The histograms depict mean (±S.E.) values in each case; results were analyzed by Student’s *t*-test. ****P<0.0001. (**d**) Activation of H2AX in vital organs and PBMCs following i.v. injection of dead and live B16-F10 cells into mice and their prevention by CNPs, DNase I and R-Cu. One hundred thousand B16-F10 cells were injected i.v. and animals killed after 24 h. The experiments were done in duplicate and 1000 cells were analyzed in each case for calculating MFI. The histograms depict mean (±S.E.) values in each case; results were analyzed by Student’s *t*-test. *P<0.05, **P<0.01.

In order to investigate whether microarray results translated into activation of DDR proteins, we initially performed a time-course analysis of dynamics of DDR activation following co-cultivation of NIH3T3 cells with dead Jurkat cells using *γ*-H2AX as an end point. We detected upregulation of H2AX, which was maximally seen at 6 h and declined gradually thereafter but did not reach baseline levels even after 144 h (Figure 6a). We show that H2AX activation could be highly significantly inhibited (*P*<0.0001) in the presence of cfCh degrading/neutralizing agents namely, CNPs, DNase I and R-Cu indicating that cfCh was directly responsible for H2AX activation (Figure 6b; Supplementary Figure 7). H2AX activation was also not seen when live Jurkat cells were used in these co-cultivation experiments (Figure 6b; Supplementary Figure 7). Having ascertained the dynamics of H2AX activation that peaked at 6 h, we looked for expression of other DDR pathway proteins at 6 h that included ATM, ATR, MDC1, p-p53, p-p21, GADD34, Nibrin, Rad50, Mre11, as well as those involved in DNA repair by non-homologous end joining, namely, DNA-PKcs, DNA ligase IV. All the above DDR-related proteins were highly significantly activated (*P*<0.01* *to* P*<0.0001) in NIH3T3 cells following co-cultivation with dead Jurkat cells (Figure 6c; Supplementary Figure 8). Co-cultivation of NIH3T3 cells with live Jurkat cells failed to activate DDR proteins (Figure 6c; Supplementary Figure 8).

#### *In vivo* experiments

We next examined if cfCh emanating from dead and live B16-F10 cells could activate systemic DDR in vital organs when injected intravenously into mice. We clearly found marked elevation of H2AX activation following injection of dead B16-F10 cells, which could be dramatically inhibited when animals were concurrently treated with cfCh degrading/neutralizing agents namely, CNPs, DNase I and R-Cu (Figure 6d; Supplementary Figure 9). Significantly, although live cells had failed to activate DDR *in vitro*, they were capable of activating H2AX *in vivo,* albeit to a significantly lesser extent, than that by dead B16-F10 cells (Figure 6d). This finding again indicated that cancer cells undergo extensive cell death following intravenous injection into mice.^25,26^

### Genomic integration of cfCh

As we had earlier proposed that activation of DDR by chromatin fragments isolated from serum of cancer patients was a critical factor in facilitating their genomic integration,^19^ we investigated whether cfCh that had emerged from dead Jurkat cells and had activated DDR in bystander NIH3T3 cells could integrate into their genomes?

#### FISH analysis

**Figure 7:**
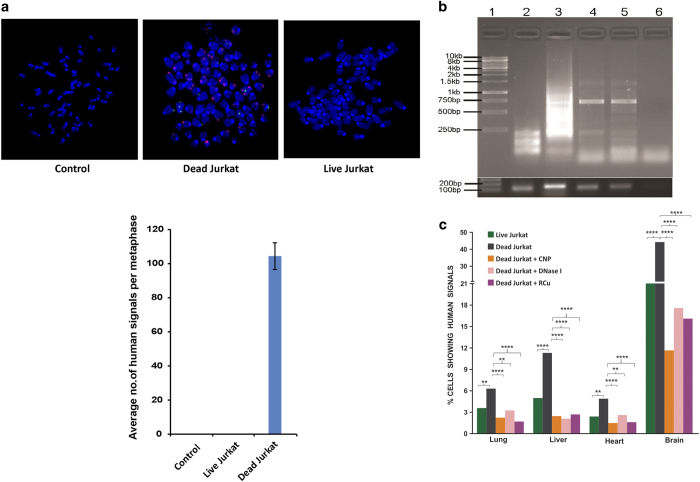
Genomic integration of cfCh *in vitro* and *in vivo* analyzed by FISH and PCR. (**a**) Representative FISH images showing presence of abundant human whole-genomic signals in metaphase spreads prepared from NIH3T3 cells co-cultivated with dead Jurkat cells at tenth passage (upper panel). No signals were detected when live Jurkat cells were similarly used. The human whole-genomic probe used did not cross react with mouse DNA. Quantitative analysis of number of human signals per metaphase (lower panel). Thirty metaphases were analyzed in each case. The figure represents mean values (±S.E.). (**b**) Gel image of PCR-amplified products showing human pan *Alu* elements in clones E7 and B10. Upper panel, lane 1: 1 kb ladder; lane 2: NIH3T3 cells (negative control); lane 3: human DOK cells (positive control); lane 4 and 5: E7 and B10 clones (respectively); lane 6: no template control. Lower panel, input control PCR, mouse *β-ACTIN* (104 bp) and human *HER2* gene (136 bp) were amplified in mouse NIH3T3 clones and human DOK cells, respectively. (**c**) Detection of human DNA signals by FISH in vital organs of mice following i.v. injection of dead and live Jurkat cells and their prevention by concurrent treatment with CNPs, DNase I and R-Cu. One hundred thousand Jurkat cells were injected i.v. and animals killed after 7 days. Two animals were used in each case and 500 nuclei were examined for each organ. Percent cells showing human signals were recorded and mean values were compared by *Χ* ^2^-test. **P<0.01, ****P<0.0001. The human whole-genomic probe used did not cross react with mouse DNA.

NIH3T3 cells co-cultivated with dead and live Jurkat cells were allowed to grow and metaphase spreads were prepared from them at tenth passage. FISH analysis on the metaphase preparations using human whole-genomic probes detected abundant positive signals indicating that human DNA from dead Jurkat cells had incorporated themselves into the genomes of NIH3T3 cells (Figure 7a, upper panel). We did not detect any FISH signals in metaphase spreads prepared from NIH3T3 cells co-cultivated with live Jurkat cells. A quantitative estimation of number of human signals per metaphase is shown (Figure 7a, lower panel).

#### Detection of human Alu elements

In order to confirm our results obtained with FISH, we looked for presence of human *Alu* sequences in two single-cell clones (E7 and B10) developed from NIH3T3 cells that had been co-cultivated with dead Jurkat cells using bioinformatics analysis. A total of 121 human *Alu* elements represented by 8 unique human *Alu* families in E7, and a total of 88 human *Alu* elements represented by 7 unique human *Alu* families in B10 were detected (Supplementary Table 4). We validated our results with PCR using pan *Alu* consensus primers, and found amplified fragments ranging from 250 bp to 1 kb with a prominent band ~750 bp in size (Figure 7b). These bands were not amplified in genomic DNA extracted from NIH3T3 cells (negative control). As expected, and consistent with earlier reports,^27^ pan *Alu* primers generated a smear in the human DOK cells (lane 3; positive control). Mouse *β-ACTIN* and human *HER2* gene were used as input controls for mouse and human genomic DNA, respectively (Figure 7b, lower panel).

#### Genomic integration of cfCh *in vivo*

We next examined if cfCh released from intravenously injected cancer cells could integrate into genome of vital organs of mice. Both dead and live Jurkat cells were intravenously injected into Balb/C mice, which were killed on day 7. FISH analysis using human-specific whole-genomic and pan-centromeric probes detected many human DNA signals in nuclei of vital organs of mice injected with both dead and live Jurkat cells (Figure 7c; Supplementary Figures 10A and B). However, the number of human signals detected in case of injected dead cells was significantly higher than those detected with respect to injection of live cells (Figure 7c). These data again suggest that live cancer cells undergo cell death following intravenous injection and release cfCh that have the ability to integrate into genomes of target organs. Once again, we show that concurrent injection of cfCh degrading/neutralizing agents namely, CNPs, DNase I and R-Cu significantly reduce the number of human signals detected (*P*<0.01 to *P*<0.0001; Figure 7c).

### Activation of inflammation

#### *In vitro* studies

**Figure 8:**
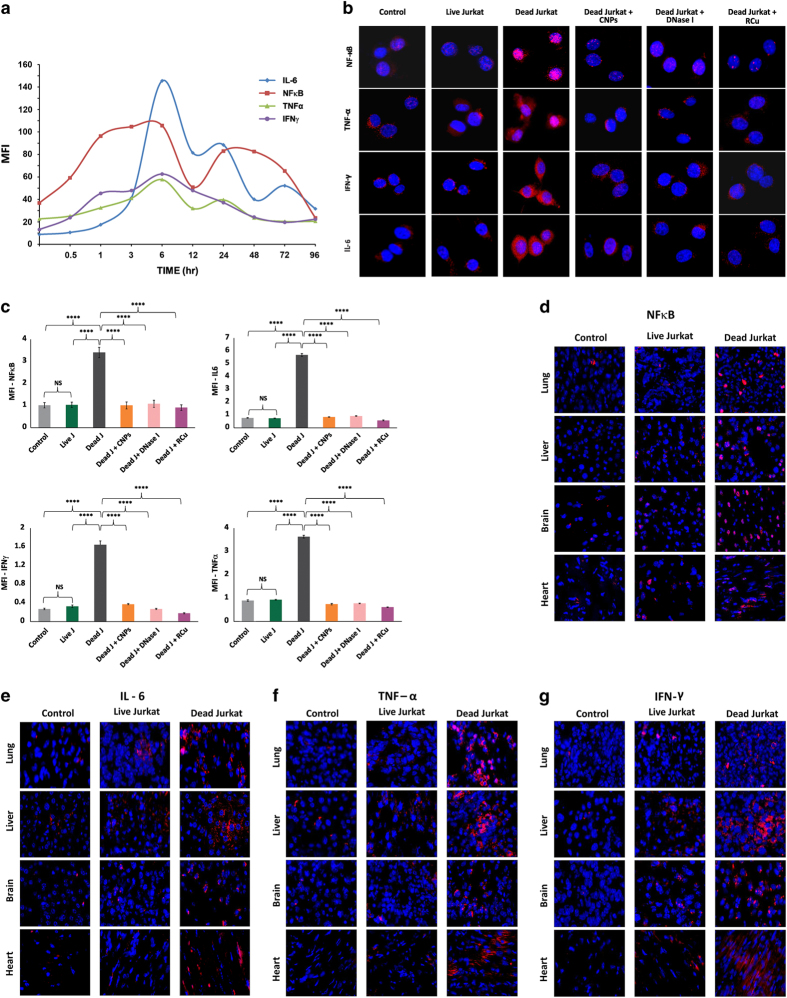

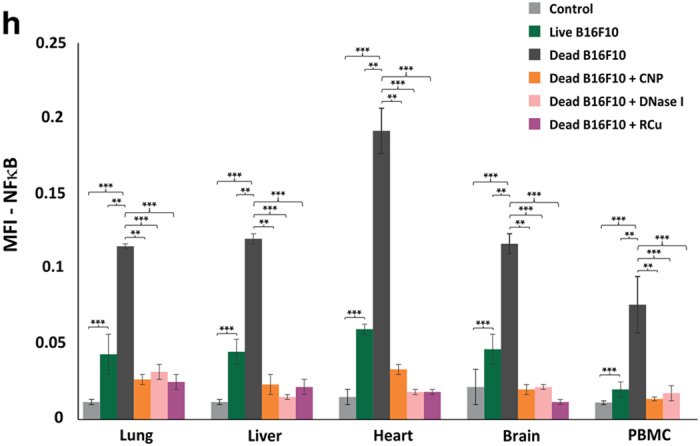
Activation of inflammation *in vitro* and *in vivo* and its prevention by chromatin neutralizing/degrading agents. (**a**) Time-course analysis of activation of inflammatory cytokines following co-cultivation of NIH3T3 cells with dead Jurkat cells as detected by indirect immunofluorescence. The experiments were done in duplicates at each time point and the mean values were plotted in the graph. (**b**) Representative images showing activation of inflammatory cytokines in NIH3T3 cells following co-cultivation with dead and live Jurkat cells with NIH3T3 cells at 6 h and their prevention by CNPs, DNase I and R-Cu. (**c**) Quantitative analysis of images shown in **b**. Activation of NF*κ*B (left upper panel), IL-6 (right upper panel), IFN*γ* (left lower panel) and TNF*α* (right lower panel). The experiments were done in duplicate and 500 cells were analyzed in each case to determine mean fluorescence intensity (MFI). Mean (±S.E.) values are depicted in the histograms and the results were compared by Student’s *t*-test. ****P<0.0001. (**d**–**g**) Representative images showing activation of inflammatory cytokines at 72 h in vital organs of mice following intravenous injection of dead and live Jurkat cells (1×10^5^) as detected by indirect immunofluorescence. (**h**) Quantitative analysis of expression of NF*κ*B following intravenous injection of dead and live B16-F10 cells (1×10^5^) and their prevention by CNPs, DNase I and R-Cu. The experiment was done in duplicate and 1000 cells were analyzed in each case to determine MFI. Mean (±S.E.) values are depicted in the histograms and the results were compared by Student’s *t*-test. **P<0.01, ***P<0.001.

In order to investigate whether microarray results using Jurkat cells that showed upregulation of inflammatory pathways translated into activation of pro-inflammatory cytokines, we initially performed a time-course analysis to examine the induction of NF*κ*B, IL-6, TNF*α* and IFN*γ* in NIH3T3 that were co-cultivated with dead Jurkat cells (Figure 8a). We detected upregulation of all four cytokines albeit with different kinetics for up to 96 h; in general, maximal activation was seen at around 6 h for all four cytokines (Figure 8a). We chose this time point (6 h) to examine if cfCh degrading/neutralizing agents namely, CNPs, DNase I and R-Cu would inhibit their activation. Figure 8b clearly shows that all three agents were highly effective in preventing upregulation of NF*κ*B, IL-6, TNF*α* and IFN*γ* in NIH3T3 cells co-cultivated with dead Jurkat cells (*P*<0.0001). Co-cultivation of live Jurkat cells failed to activate inflammatory cytokines (Figure 8b). These findings are quantitatively depicted in Figure 8c.

#### *In vivo* studies

When injected intravenously, dead Jurkat cells induced an intense inflammatory response involving NF*κ*B, IL-6, TNF*α* and IFN*γ* in lung, liver, brain and heart of mice at 72 h (Figures 8d–g). Injection of live Jurkat cells also activated the above cytokines albeit to a lesser degree (Figures 8d–g). We next conducted experiments using dead and live B16-F10 melanoma cells and observed similar systemic activation of inflammatory cytokines (Supplementary Figure 11). Quantitative analysis given in Figure 8h shows that the three chromatin neutralizing/degrading agents dramatically inhibited this inflammatory reaction in all organs examined as well as in PBMCs. The figure also shows that live B16-F10 cells were capable of activating NF*κ*B but to a lesser degree.

## Discussion

Transfer of DNA from dead cells in co-cultivation experiments have been reported previously.^28–30^ Bergsmedh *et al.* observed that apoptotic bodies that were released from H-ras(V12) and human c-myc-transfected rat fibroblasts were phagocytosed by *p53*^*−*^*/p53*^*−*^ mouse cells and FISH could detect presence of rat or rat–mouse fusion chromosomes in their nuclei. Genomic integration of phagocytosed DNA led to oncogenic transformation of *p53*^*−*^*/p53*^*−*^ mouse cells.^28^ Although induction of DNA damage and inflammation was not investigated in this study, we show here that genomic integration of cfCh released by dying cancer cells can directly trigger both DDR and inflammation in the bystander healthy cells both *in vitro* and *in vivo*, which can potentially lead to their oncogenic transformation (Figures 2f, 3a and b). We also show that DDR and inflammation are closely linked pathologies and are concurrently activated, and co-expressed by the same bystander cells both *in vitro* and *in vivo* thereby creating within them an intracellular milieu that is pro-oncogenic (Figure 2f, middle and lower panels, Figure 4; Supplementary Figure 4). Our results also highlight the rapidity and extent to which cfCh can infiltrate bystander cells; near maximal uptake of cfCh could be achieved as early as 1 h and innumerable cfCh particles could be seen entering their nuclei (Supplementary Figure 2). Nuclear accumulation of cfCh was followed by their genomic integration in host cells as detected by FISH and the presence of multiple unique human *Alu* elements in the recipient cells (Figures 7a–c). Genomic integration of cfCh resulted in genome-wide deregulation of transcription involving 1004 genes (Figure 5a; Supplementary Table 2) including upregulation of multiple pathways related to phagocytosis, DNA damage and inflammation (Figures 5b–d; Supplementary Table 3). Surprisingly, while live cancer cells when co-cultivated with NIH3T3 cells *in vitro* did not release cfCh (Figures 1a and b), and consequently did not activate DDR and inflammation in the bystander cells (Figures 6b, c and 8b, c), when injected intravenously into mice, live cancer cells underwent active cell death and released cfCh into bystander cells of distant target organs to induce *γ*H2AX and NF*κ*B, IL-6, IFN*γ* and TNF*α* (Figure 6d, 8d–h; Supplementary Figures 9). When injected *in vivo*, the incoming cfCh from both dead and live cells integrated themselves into genomes of cells of target organs, which was detectable by FISH (Figure 7c). In this regard, injected live cells behaved in a manner similar to dead cells having undergone apoptosis upon reaching distant target organs. Both dead and live cells exhibited co-localization of *γ*H2AX and NF*κ*B in the same bystander cells of brain, liver and lung when injected *in vivo* suggesting the creation of a pre-cancerous milieu in such cells (Figure 4).

DNA damage and inflammation are hallmarks of cancer and act synergistically to drive the oncogenic process.^11,31^ It has been proposed that DNA damage-induced mutations and genomic instability acting in tandem with inflammation can orchestrate a ‘perfect storm’ in the microenvironment of a tumor, which can lead to oncogenic transformation of the resident bystander cells.^9^ Our results show that cfCh from dead cancer cells are responsible for both DNA damage and inflammation, and that they are often co-activated in the same cells suggesting that such cells have a high potential for oncogenic transformation. Thus cfCh released from dying cancer cells may recruit bystander cells of the microenvironment into the oncogenic process thereby promoting local tumor invasion. cfCh may also play a key role in cancer metastasis as many circulating tumor cells (CTCs) are apoptotic in nature,^32,33^ which may lodge in distant organs to induce bystander oncogenesis in target cells thereby generating new cancers in host tissues that could masquerade as metastasis. Supportive evidence that dead CTCs might be involved in metastasis comes from ‘surprising’ observations that patients with breast cancer whose CTCs are enriched in apoptotic cells have a particularly poor prognosis,^33^ and in patients with colorectal cancer, apoptotic CTCs are associated with liver metastasis.^34^ It has been further suggested that different forms of cancer treatment namely, surgery, radiotherapy and chemotherapy could all mobilize CTCs into circulation thereby promoting metastatic spread of cancer.^35^ Our observation that cfCh degrading/neutralizing agents namely, CNPs, DNase I and R-Cu can abrogate bystander DNA damage and inflammation both *in vitro* and *in vivo* suggests therapeutic possibilities to prevent local and systemic spread of cancer.

## Materials and methods

### Cell lines and induction of cell death

We used three different cancer cell lines: B16-F10 (mouse melanoma), Jurkat (human lymphoblastic leukemia) and HeLa (human cervical cancer). The HeLa cells stably express a GFP fusion to the Golgi protein N-acetylgalactosaminyltransferase-2 (GalNAc-T2-GFP).^36^ The normal cell line used was NIH3T3 (mouse fibroblast). The sources and their culture conditions are given in Supplementary Table 1.

NIH3T3 cells were seeded at a density of 6×10^4^ cells in 35 mm^3^ culture dishes in 1.5 ml of DMEM+bovine calf serum (10%); after 16 h (cell count ~10×10^4^), dead donor cells were usually added to them in a ratio of 1 : 1 unless specified otherwise. Cell death was induced in B16-F10 and Jurkat cells by treating them with adriamycin (5 *μ*g/ml for 48 h) (Pfizer Products India Pvt. Ltd., Mumbai, India) or activated anti-Fas ligand (6 *μ*g/ml for 24 h) (Millipore, Temecula, CA, USA; Catalog No. 05-201). Cell death was confirmed when >95% of the population was excluded by Trypan blue dye assay. After specified time periods the dead cells were collected, washed three times in 5 ml phosphate buffered saline (PBS) by centrifugation (600×*g *for 5 min) and the number of dead cells in the pellets was counted. Cell death in GalNAc-T2-GFP HeLa cells was induced by administering radiation from a Cobalt source (15 Gy). We also used live B16-F10, Jurkat and GalNAc-T2-GFP HeLa cells in control experiments. They were similarly washed, centrifuged and the cell pellets were used in co-cultivation experiments.

### Co-cultivation experiments

Two different co-cultivation experiments were performed.

1. Co-cultivation of NIH3T3 cells with dead and live Jurkat cells: Jurkat cells (5×10^6^) were labeled with 13 *μ*M BrdU (Sigma Chemicals, St Louis, MD, USA; Catalog No. B5002-100MG) for 24 h and induced to undergo cell death by treatment with anti-FAS ligand (24 h) or adriamycin as described above. Dead Jurkat cells were co-cultivated with NIH3T3 cells in a ratio of 1 : 1 in 35 mm^3^ petri dishes for specified time periods. Cells were thoroughly washed and processed for confocal microscopy, fluorescence microscopy or microarray analysis.
2. Co-cultivation of NIH3T3 cells with dead and live GalNAc-T2-GFP HeLa cells: GalNAc-T2-GFP HeLa cells were labeled with BrdU (25 *μ*M for 36 h), washed, trypsinzed and seeded at a density of 5×10^3^ on cover slips in 35 mm^3^ petri dishes for 12 h allowing cells to attach to the cover slips. To induce cell death, they were administered ionizing radiation (15 Gy) and were immediately overlaid with 1×10^5^ NIH3T3 cells (1 : 20) and cultured for 36 h. Cells were thoroughly washed and processed for fluorescent microscopy for detection of BrdU signals and activation of H2AX, p-ATM, NF*κ*B and active Caspase-3 in bystander NIH3T3 cells.

### *In vivo* experiments

C57/BL6 mice (6–8 weeks old weighing ~20 g) obtained from and housed in the Institute Animal House Facility. The protocol for the experiment was approved by the Institutional Animal Ethics Committee (IAEC) of the Institute. Mice were intravenously injected with dead or live BrdU-labeled B16-F10 melanoma or Jurkat cells (10×10^4^) and were killed after 24 or 72 h as specified by cervical dislocation and their vital organs and PBMCs removed, and processed for detection of fluorescent BrdU signals and activation of H2AX, NF*κ*B, IL-6, TNF*α* and IFN*γ*. Control mice received saline injection. In some experiments, free BrdU was injected into mice in the same concentration that we normally use for labeling cultured cells *in vitro* (13 *μ*M) after equating for blood volume of mice (5 ml).

### Confocal microscopy

#### Co-cultivation of NIH3T3 cells with BrdU-labeled dead and live Jurkat cells

NIH3T3 cells grown on cover slips were co-cultivated with BrdU pre-labeled dead and live Jurkat cells in a ratio of 1 : 1. In some experiments chromatin neutralizing/degrading agents namely, CNPs, DNase I and R-Cu was added to the co-culture of dead Jurkat cells with NIH3T3 cells. The cover slips were collected at various time points as specified, washed in PBS, fixed with 4% paraformaldehyde for 30 min, washed thrice with PBS, treated with 0.02% Triton-X 100 for 30 min. This was followed by treatment with 2 M HCl for 1 h and washing three times with PBS and glycine. Cells were then treated with 3% BSA for 1 h and immuno-stained with anti-BrdU antibody (1 : 100 dilution) (Abcam, Cambridge, MA, USA; Catalog No. ab6326) overnight at 4 °C. Dylight-550 was used as secondary antibody (1 : 500 dilution) (Abcam, Catalog No. ab98387) for 1 h, mounted onto clean glass slides with Vecta-shield and stained with DAPI. The stained nuclei were visualized using Zeiss differential laser scanning confocal microscopy platform. Fifty nuclei were randomly chosen for analysis and the mean nuclear fluorescence intensity was measured using LSM Image Examiner 4.0 software (Carl Zeiss Jena GmbH, Oberkochen, Germany).

### Microarray analysis

NIH3T3 cells were co-cultivated with dead Jurkat cells and microarray analysis performed as described below.

#### Data filtering using statistical parameters

Expression array hybridization using the Agilent labeling kit (Agilent’s Quick-Amp labeling Kit 5190-0442, Agilent Technologies, Santa Clara, CA, USA), Agilent hybridization kit (5188-5242, Agilent Technologies) and mouse GXP_8X60K slides were carried out by Genotypic Technology Private Ltd, Bengaluru, India according to the manufacturer’s protocol. Agilent Feature extraction software (Agilent Technologies) was used to extract raw data from image files followed by essential quality control steps. The data structure comprises of three samples in duplicates: NIH3T3_Control_(0 h), NIH3T3+dead Jurkat_(2 h) and NIH3T3+dead Jurkat_(6 h). All the pre-processing steps (background correction, normalization, probe filtration, etc) of the gene expression data were carried out using Agi4x44PreProcess Bioconductor package in R. For background correction and normalization between arrays, ‘backgroundCorrect (half)’ and ‘normalizeBetweenArrays (quantile)’ functions from the limma package were, respectively, used. To check variability across samples all data were log transformed and median centered. To exclude non-differential genes and to limit analysis only for significantly differential genes, class comparison was carried out between all possible groups using BRB array software.^37^ Class comparison between groups of arrays was based on univariate parametric test and inclusion of genes was based on *P*-values <0.05. Heat maps were generated using MeV software v4.9.0.^38^ GSEA analysis^39^ using KEGG gene set (http://www.genome.jp/kegg/pathway.html) was performed to identify underlying biological processes and pathways. This open source web-based tool uses a database of biological information curated from the literature.

### Detection of DDR and inflammatory cytokines by indirect immunofluorescence

#### *In vitro* studies

NIH3T3 cells were co-cultivated with dead and live Jurkat cells for 6 h as described earlier. Methodology for detection of activated H2AX and other DDR proteins by indirect immunofluorescence including sources of reagents have been described by us earlier.^19^ Activation of inflammatory cytokines was similarly analyzed by indirect immunofluorescence using specific antibodies after 6 h of co-cultivation. The antibodies used and their sources are given in Supplementary Table 5. Nuclear fluorescence of NF*κ*B and cell-associated fluorescence of IL-6, TNF*α*, IFN*γ* were quantified using Image J Software (Wayne Rasband, National Institute of Mental Health, Bethesda, MD, USA). Experiments were done three times in duplicates; 500 cells were analyzed in each case and the mean of the three experiments was determined.

#### *In vivo* studies

C57/BL6 mice (6–8 weeks old weighing ~20 g) were injected intravenously with dead and live Jurkat or B16-F10 cells (10×10^4^) as specified in 100 *μ*l of saline; control mice were injected with saline alone. Two mice were used for treatment in each group. Animals were killed for collection of blood and removal of vital organs after 24 or 72 h as specified. The cryosections were processed for immunofluorescence for *γ*H2AX was as described by us earlier,^19^ whereas induction of inflammatory cytokines was detected by indirect immunofluorescence as described above. For quantification of NF*κ*B activation, at least 1000 cells were analyzed from 10 randomly chosen fields for each tissue for each mouse and 100 nuclei per animal were analyzed in case of PBMCs. The average number of nuclei exhibiting positive fluorescence was recorded using Image J software and the percentage figures calculated.

### Experiments using cfCh degrading/neutralizing agents

Pullulan-histone antibody nanoconjugates (CNPs) were synthesized as described in our recent publication using H4 IgG.^19,40^ CNPs have been shown to specifically bind to chromatin fragments and inactivate them both *in vitro* and *in vivo. In vitro* experiments were performed on cells grown at a density of 10×10^4^ and co-cultivated with dead Jurkat cells for 6 h (unless specified otherwise) in the presence or absence of CNPs (5 *μ*g H4 IgG/ml). The treated cells were analyzed with respect to various parameters as described in the text. *In vivo* experiments were conducted by injecting mice intravenously with a single dose of dead cells (10×10^4^ cells) through tail vein with and without concurrent injection of CNPs (50 *μ*g H4 IgG/mouse i.p.). Treatment with CNPs was started 4 h prior to the administration of dead cells; and every 24 h thereafter. Animals were killed at specified times and their vital organs, namely, heart, lung, liver, brain as well as PBMCs were removed for analysis of different parameters as mentioned above.

#### DNase I

Bovine pancreatic DNase I was obtained from Sigma Aldrich (St Louis, MO, USA) (Catalog No. DN25-1G). About 0.05U of DNase I per 1.5 ml of culture media was used for *in vitro* experiments. Fifteen mg/kg of DNase I was administered i.p. *in vivo* experiments. The treatment of DNase I was started 4 h prior to administration of dead cells and every 12 h thereafter.

#### Resveratrol–copper

R is a widely investigated plant polyphenol with antioxidant properties.^41^ However, R acts as a pro-oxidant in the presence of Cu by its ability to reduce Cu (II) to Cu (I) thereby generating a free radical.^42^ R-Cu has been shown to be capable of cleaving plasmid DNA via this pro-oxidant property.^43^ We have recently reported that R-Cu can also degrade genomic DNA,^44^ and that the pro-oxidant property of R-Cu with respect to DNA degradation can be retained even when the molar concentration of Cu is reduced more than 1000-fold with respect to that of R.^44^ The R-Cu dose used in our *in vitro* experiments was at a molar ratio of 1 mM R : 0.0001 mM Cu. Resveratrol (2.3 mg) (Sigma, St Louis, CA, USA; Catalog No. R5010) was dissolved in 5 ml of 60% ethanol (2 mM) (solution A). Copper sulfate (4.98 mg) (MP Biomedicals, Illkirch, France; Catalog No. 191415) was dissolved in 1 ml distilled water (20 mM) and then serially diluted to 0.0002 mM concentration (solution B). About 50% v/v of solution A and solution B was mixed to obtain a mixture containing 1 mM R and 0.0001 mM Cu. One hundred  *μ*l of this mixture was added to 1.5 ml of culture media. For *in vivo* experiments, we used resveratrol and copper that have been approved for human use (TransMaxTR, Biotivia LLC, Arlington, VA, USA and Chelated Copper, J.R. Carlson Laboratories Inc., Arlington Heights, IL, USA). Contents of R capsules (500 mg) were dissolved in distilled water (0.4 mg/ml); chelated copper tablets (5 mg) were crushed into fine powder and dissolved in distilled water (0.04 *μ*g/ml). Fifty *μ*L of both the solutions were administered to mice by oral gavage one after the other at final dose of R=1 mg/kg and Cu=0.1 *μ*g/kg, the ratio between R & Cu being maintained at ∼1: 10^−4^.

### Fluorescence *in situ* hybridization

#### *In vitro* experiments

NIH3T3 cells were co-cultivated with dead and live Jurkat cells and allowed to grow. After 10 passages the cells were processed for FISH to detect the presence of human DNA signals on metaphase spreads as described us earlier.^19^ Fifty metaphases were analyzed for presence of human genomic signals. The human whole-genomic FISH probes used were un-reactive to mouse DNA.

#### *In vivo* experiments

C57/BL6 mice (6–8 weeks old weighing ~20 g) were intravenously injected with dead and live Jurkat cells (10×10^4^ cells) and were killed on day 7 by cervical dislocation and their vital organs and PBMCs removed, and processed for FISH. Control mice received saline injection. The treated and control groups contained two mice each and FISH for detection of human signals (genomic and/or centromeric) was performed as described by us earlier.^19^ One thousand cells were analyzed per animal for each tissue from 10 randomly chosen fields and the percentage of nuclei showing human signals was recorded.

### Detection of human Alu elements

We developed two single-cell clones (E7 and B10) from NIH3T3 cells co-cultivated with dead Jurkat cells and analyzed them for the presence of human *Alu* elements by whole-genome sequencing and pan *Alu* PCR. Sequencing was carried out using Illumina GA IIX (Genotypic Technology Pvt Ltd) and detection of *Alu* elements was carried out using bioinformatic analysis.^19^ The PCR experiment was performed as follows: genomic DNA was PCR amplified using primers for human pan Alu sequences – TC-65 (5ʹ-AAGTCGCGGCCGCTTGCAGTGAGCCGAGAT-3ʹ) and 517 (5ʹ-CGACCTCGAGTTGCAGTGAGCRYAGAT-3ʹ) as described by Nelson *et al.*^27^ In brief, Genomic DNA was extracted from the cells and quantified using Nanodrop 2000c Spectrophotometer (Thermo Fisher Scientific Inc., Wilmington, DE, USA). The *pan Alu *PCR reaction was performed in 20 *μ*l volume containing 10 *μ*l (2x) Biomix-Red master mix (Bio25005), 5 *μ*M each primer, 200 ng gDNA from each sample. PCR condition was as follows: initial denaturation: 95 °C, 3 min; denaturation: 94 °C, 30 s; annealing: 55 °C, 30 s; extension: 72 °C, 5 min for 35 cycles and final extension 72 °C for 10 min. The all 20 *μ*l PCR reaction was resolved on 1.2% agarose gel containing ETBR along with 1 kb ladder. The mouse *β-ACTIN* and human *HER2* gene were used as input controls and PCR amplified using primers (OAD1487 5ʹ-CTAAGGCCAACCGTGAAAAG-3ʹ and OAD1488 5ʹ-ACCAGAGGCATACAGGGACA-3ʹ) and (OAD1099 5ʹ-GAGGCTGTGTGGTGTTTGG-3ʹ and OAD1100 5ʹ-CGTGGATGTCAGGCAGATG-3ʹ), respectively. The PCR conditions were as follows: initial denaturation: 95 °C, 3 min; denaturation: 94 °C, 30 s; annealing: 55 °C, 30 s; extension: 72 °C, 30 s for 35 cycles and final extension 72 °C for 5 min. The 20 *μ*l PCR reaction was resolved on 1.8% agarose gel containing ETBR along with 100 bp ladder.

### Statistical analysis

GraphPad Prism 5 (GraphPad Software, Inc., San Diego, CA, USA; Version 5.0) was used for statistical analysis. Data were compared using the Student’s *t-*test and by *Χ* ^2^ analysis, and as indicated in appropriate places in legends to figures.
