# Supplementary material for: Cell-free chromatin from dying cancer cells integrate into genomes of bystander healthy cells to induce DNA damage and inflammation
Source: Cell Death Discov. 2017 May 29;3:17015–. doi: 10.1038/cddiscovery.2017.15 (PMC5447133; doi:10.1038/cddiscovery.2017.15)
Supplement: Supplementary Figures [file cddiscovery201715-s1.docx]

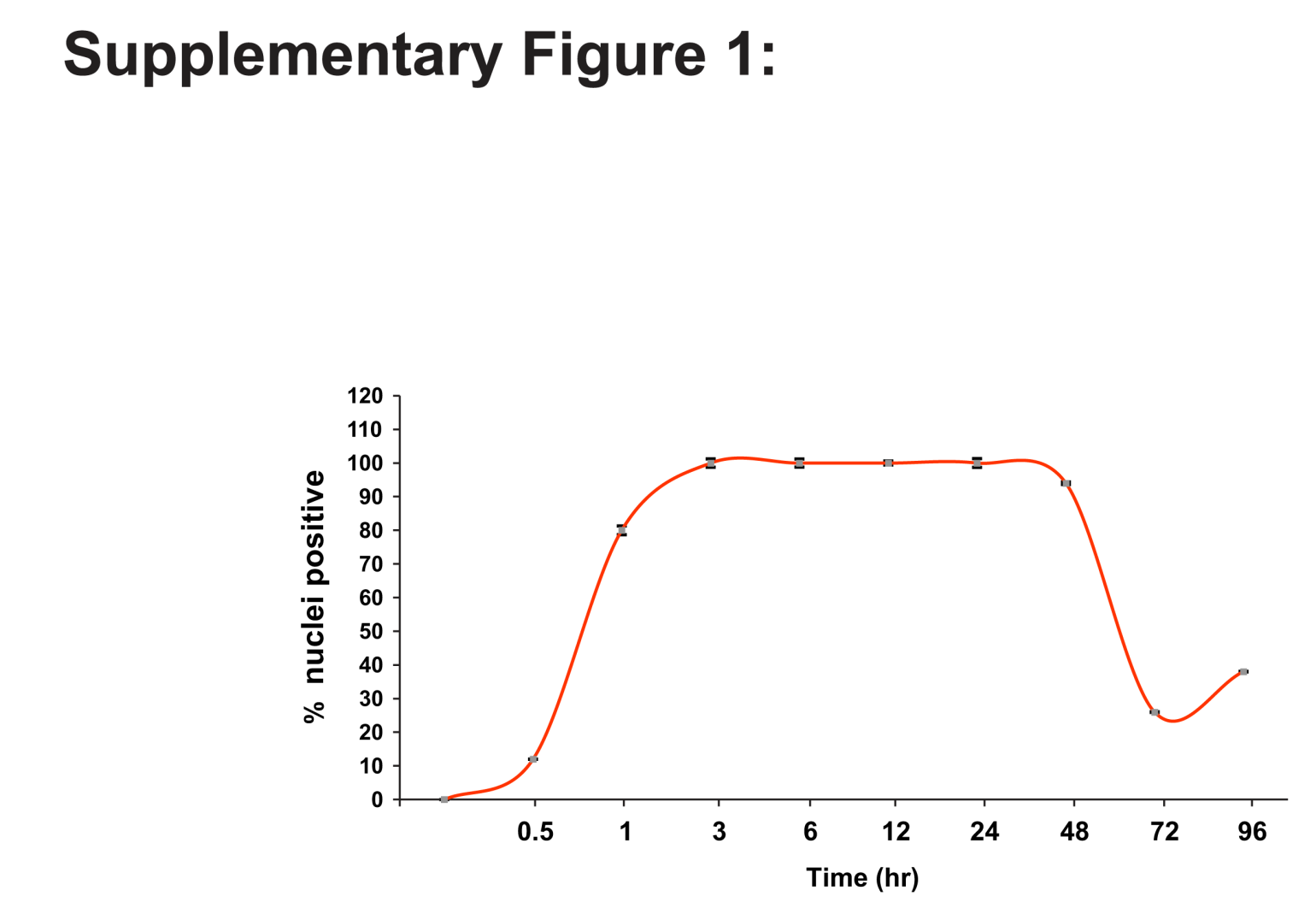


**Supplementary figure 1**

**Supplementary figure 2**

**Supplementary figure 3**


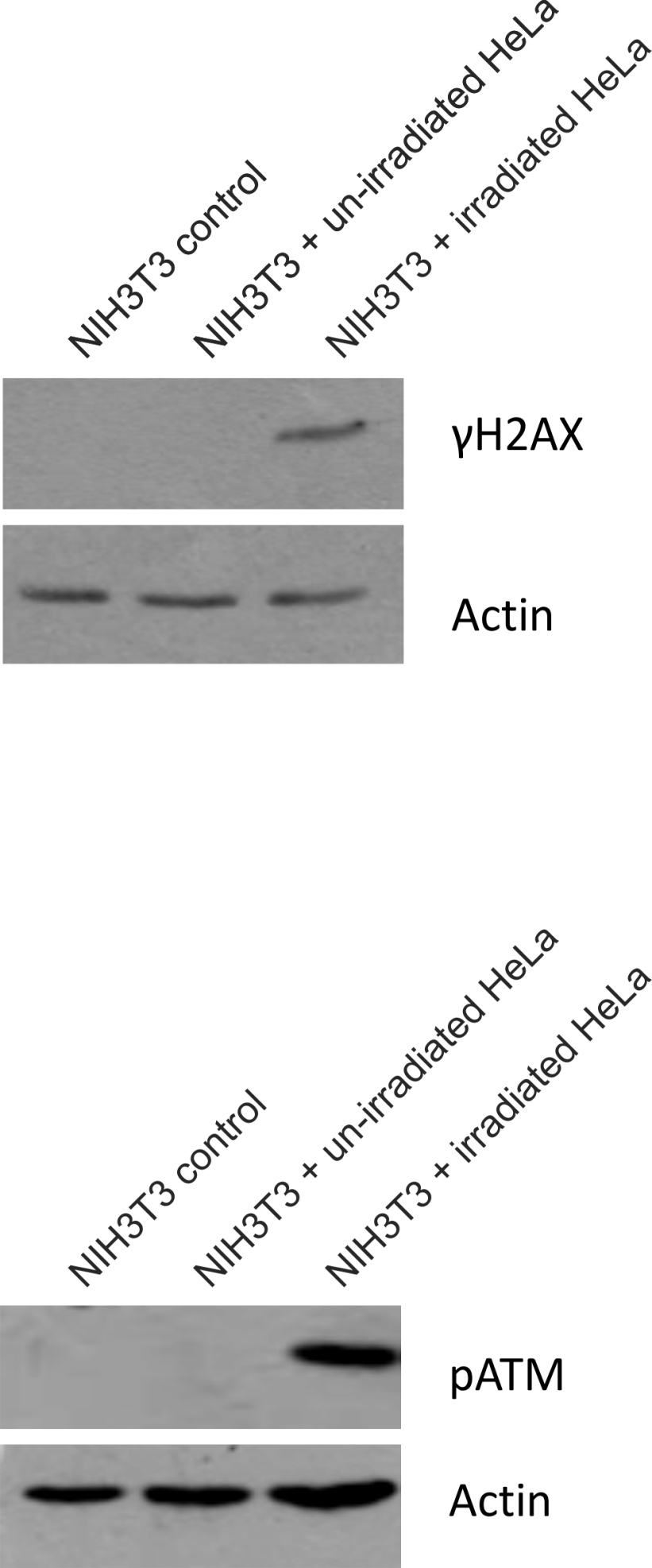


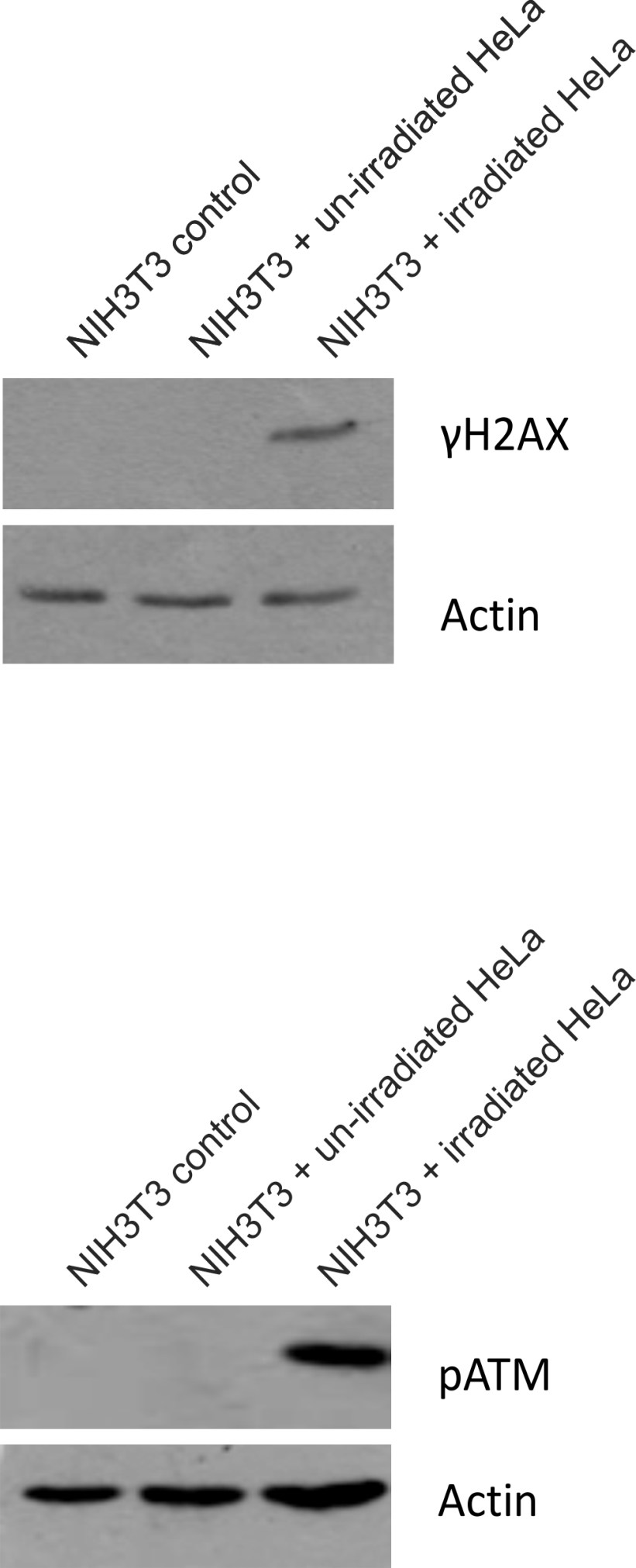


**Supplementary figure 4**


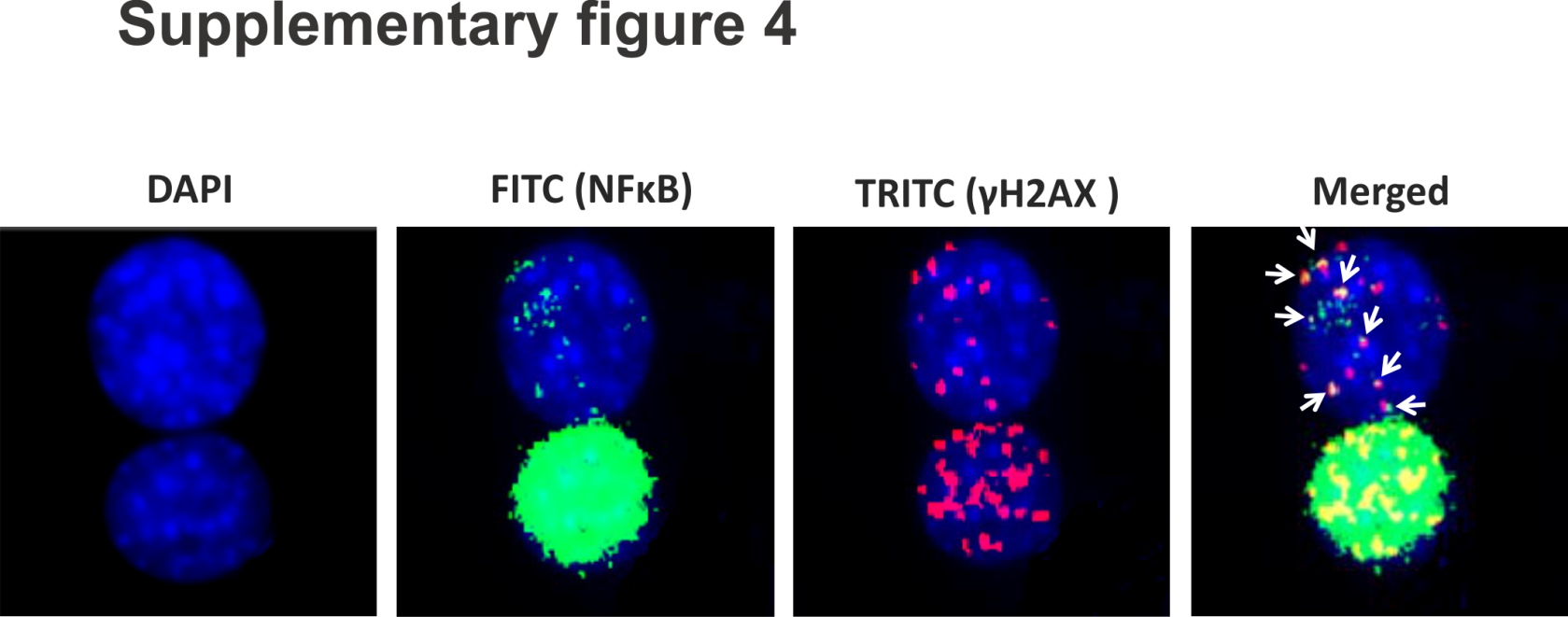


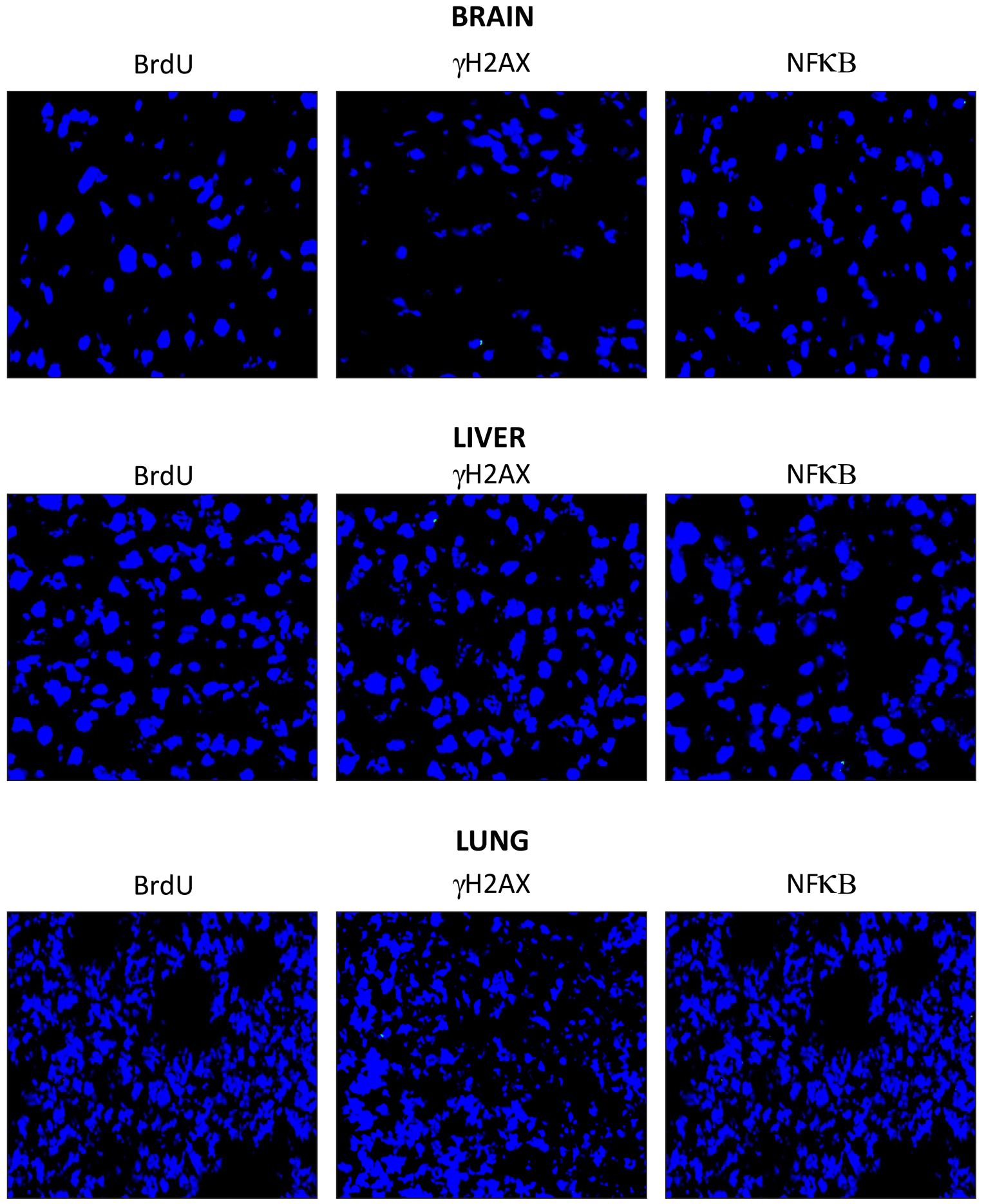


**Supplementary Figure 5**

**Supplementary figure 6**


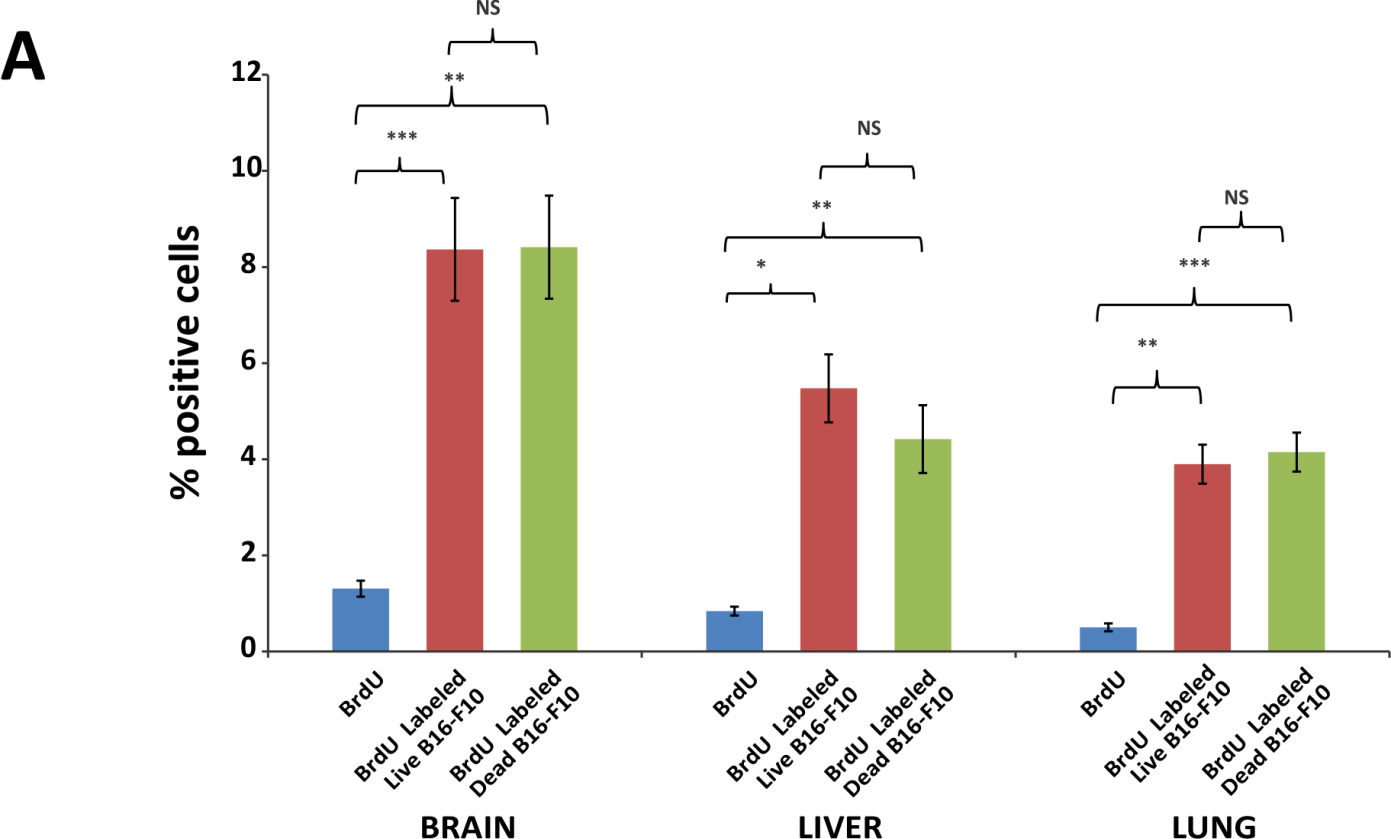


**A**


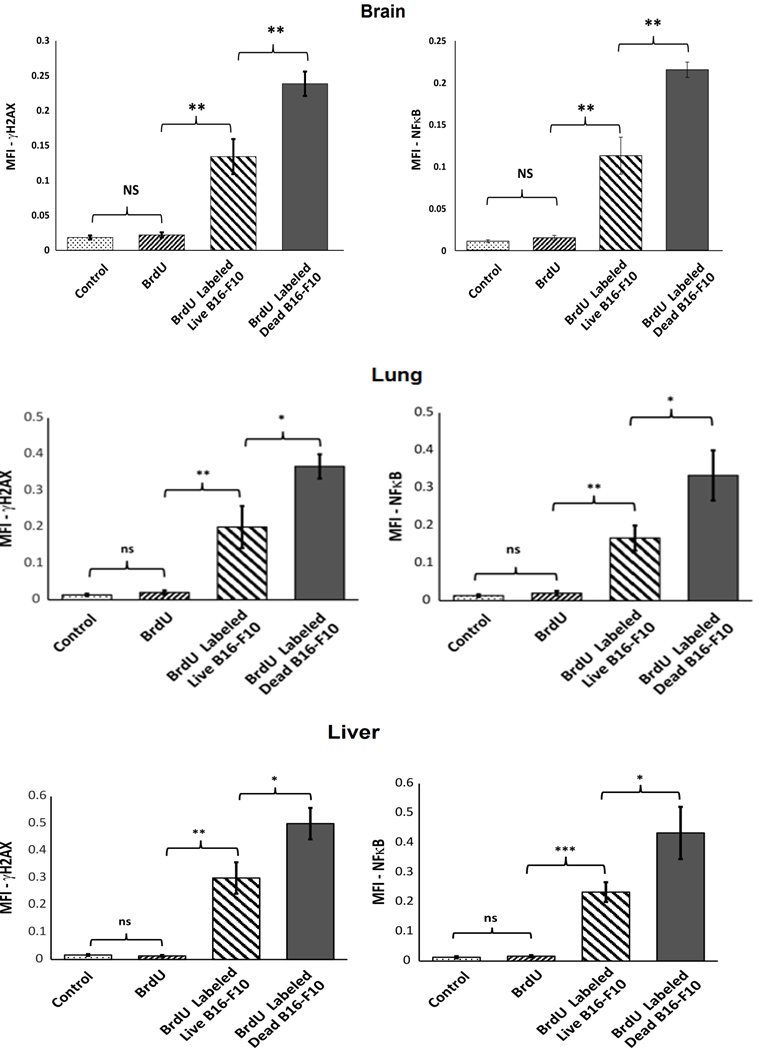


**B**

**Supplementary figure 7**

**Supplementary figure 8**


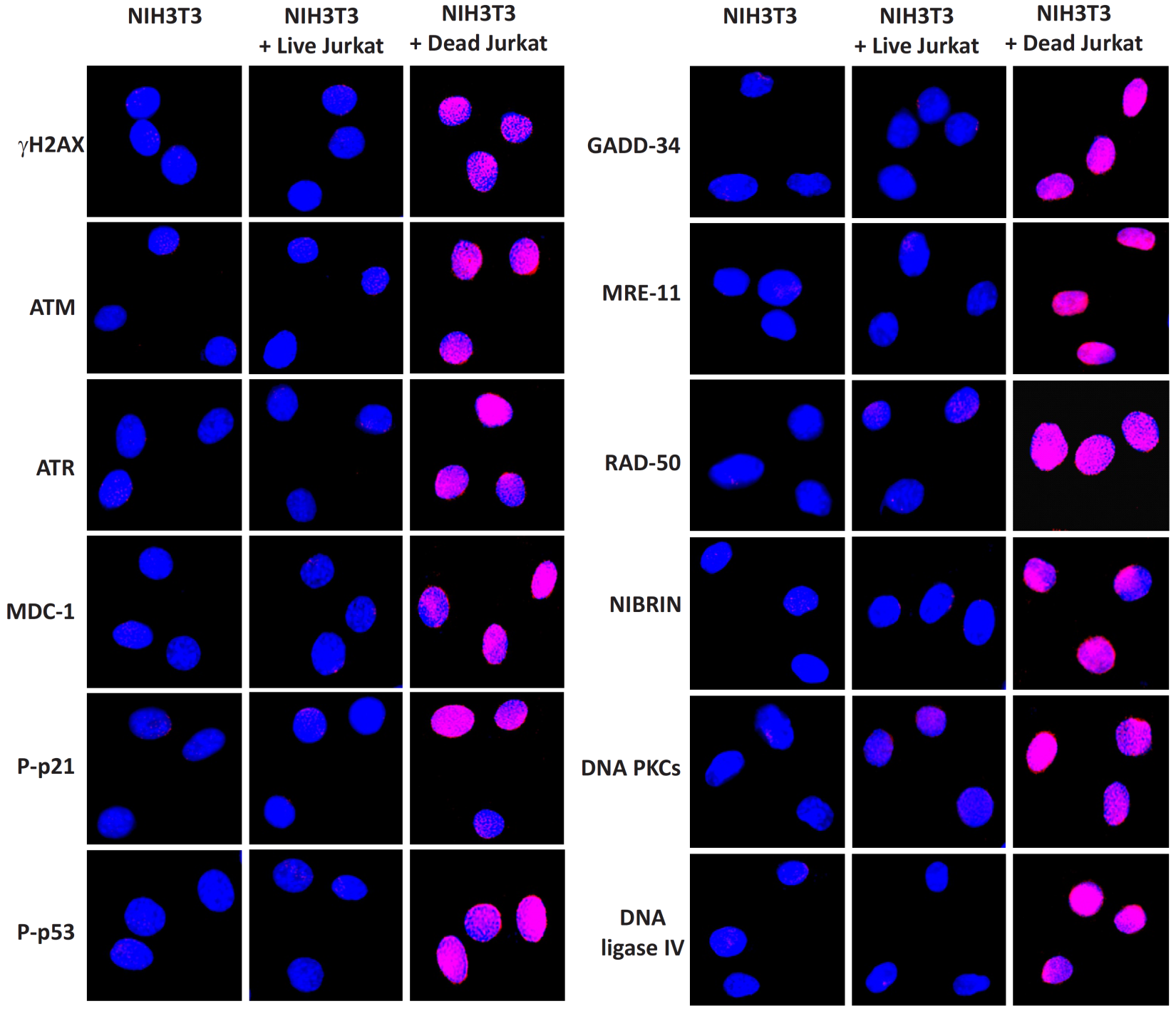


**Supplementary figure 9**


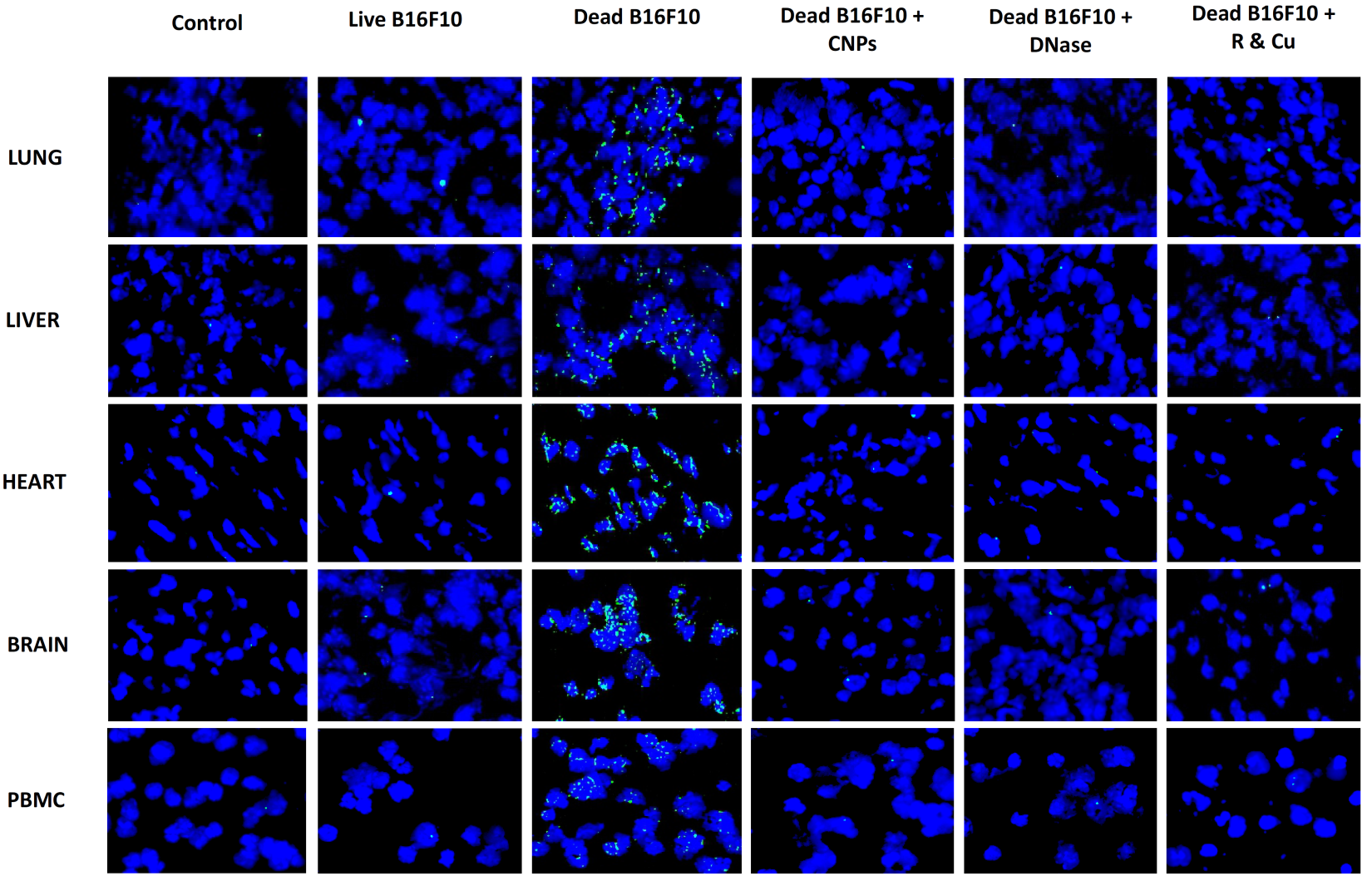


**Supplementary figure 10**


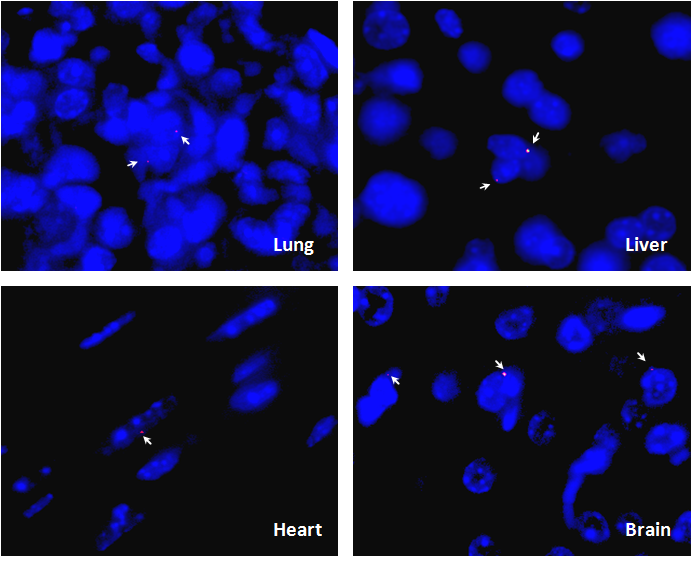

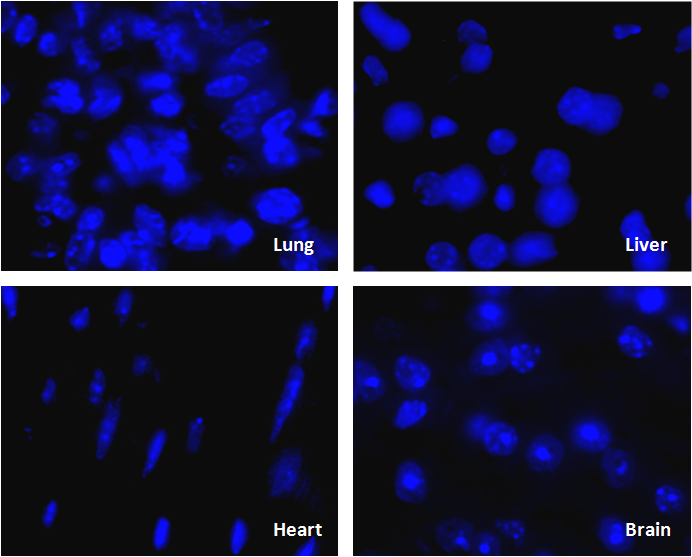


**B**

**A**


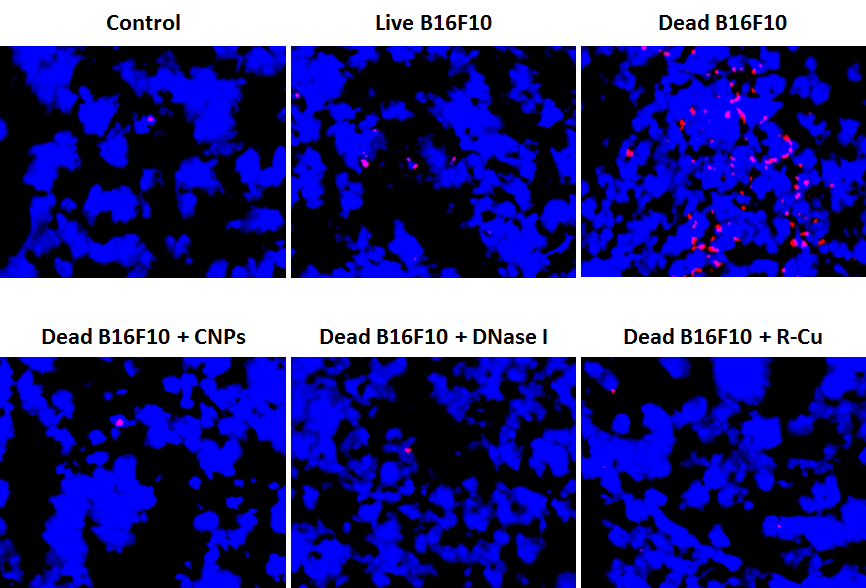


**Supplementary figure 11**
