# Supplementary material for: Cell-free chromatin from dying cancer cells integrate into genomes of bystander healthy cells to induce DNA damage and inflammation
Source: Cell Death Discov. 2017 May 29;3:17015–. doi: 10.1038/cddiscovery.2017.15 (PMC5447133; doi:10.1038/cddiscovery.2017.15)
Supplement: Supplementary Figure Legends [file cddiscovery201715-s2.docx]

**Legends to supplementary figures**:

**Supplementary figure 1**:

Inhibition of nuclear uptake of BrdU labeled cfCh by NIH3T3 cells co-cultivated with dead Jurkat cells in presence of Actinomycin-D (0.0005 μg /ml) and at low temperature (31^o^C) analyzed by confocal microscopy at 6hr. The experiments were done in duplicate and 100 randomly chosen nuclei were analyzed in each case. Results (Mean ± S.E.) were compared by Student’s *t-test*. ****p<0.0001.

**Supplementary figure 2**:

Kinetics of nuclear uptake of BrdU labeled cfCh by NIH3T3 cells co-cultivated with dead Jurkat cells as analyzed by confocal microscopy. Fifty randomly chosen cells were analyzed at each time-point and percent positive nuclei were recorded. The experiment was done in duplicate at each time point and the average values are depicted in the graph.

**Supplementary figure 3**:

Western blot analysis showing activation of H2AX (upper panel) and p-ATM (lower panel) in bystander NIH3T3 cells co-cultivated with irradiated and un-irradiated GalNAc-T2-GFP HeLa cells at 36 hr.

**Supplementary figure 4**:

Co-localization of γH2AX and NFκB fluorescent signals in bystander NIH3T3 cells co-cultivated with irradiated GalNAc-T2-GFP HeLa cells at 36 hr.

**Supplementary Figure 5**:

Control images of mice not injected with B16-F10 melanoma cells with respect to BrdU uptake and activation of γ-H2AX and NFκB in the context of experiments described in legends to Figures 3A and 3B.

**Supplementary Figure 6**:

Uptake of BrdU by vital organs of mice following injection of free BrdU and dead and live B16-F10 melanoma cells pre-labeled with BrdU and activation of H2AX and NFκB. **A**. Free BrdU (13µM) was intravenously injected into 3 mice and animals were sacrificed after 72hr. For comparison, other animals, 3 in each case, were injected with dead and live B16-F10 melanoma cells (10 x 10^4^) pre-labeled with BrdU. One thousand cells were analyzed in each case and the average number of cells showing fluorescent signals was determined. **B**. Activation of H2AX and NFκB following injection of free BrdU and dead and live B16-F10 cells pre-labeled with BrdU in brain, lung and liver of mice. Experiment was conducted as described under A and mean nuclear fluorescence intensity with respect to γH2AX and NFκB was determined. Results (Mean ± S.E.) compared by Student’s *t-test*. *p<0.05, **p<0.01, ***p<0.001; NS = not significant.

**Supplementary Figure 7**:

Representative images with respect to H2AX activation in experiments described in legends to Figure 6B using co-culture of dead and live Jurkat cells with NIH3T3 cells at 6 hr with and without cfCh neutralizing / degrading agents.

**Supplementary Figure 8**:

Representative images with respect to activation of DDR and DNA-repair proteins in experiments described in legends to Figure 6C using co-culture of dead and live Jurkat cells with NIH3T3 cells at 6 hr.

**Supplementary Figure 9**:

Representative images with respect to H2AX activation in experiments described in legends to Figure 6D using dead and live B16-F10 melanoma cells with and without cfCh neutralizing / degrading agents.

**Supplementary Figure 10**:

Representative images with respect to detection of human DNA signals by FISH in experiments described in legends to Figure 7C. **A**. Control animals show no signals. **B**. Treated animals show presence of human DNA signals in vital organs. Human whole genomic signals (green), human pan-centromeric signals (red).

**Supplementary Figure 11**:

Representative images with respect to NFκB activation in lung in experiment described in legends to Figure 8H using dead and live B16-F10 melanoma cells with and without cfCh neutralizing / degrading agents.
