# Supplementary material for: Cell-free chromatin from dying cancer cells integrate into genomes of bystander healthy cells to induce DNA damage and inflammation
Source: Cell Death Discov. 2017 May 29;3:17015–. doi: 10.1038/cddiscovery.2017.15 (PMC5447133; doi:10.1038/cddiscovery.2017.15)
Supplement: Supplementary Tables [file cddiscovery201715-s3.docx]

**Supplementary table 1:**

| **Sr. No** | **Cell line Nomenclature** | **Catalogue No.** | **Origin** | **Culture conditions used** | **Source** |
| --- | --- | --- | --- | --- | --- |
| 1 | B16-F10 | CRL-6475™ | mouse melanoma | DMEM + FBS (10%) + 1% Antibiotic cocktail | ATCC |
| 2 | Jurkat, Clone E6-1 | TIB-152™ | human lymphoblastic leukemia | DMEM + FBS (10%) + 1% Antibiotic cocktail | ATCC |
| 3 | GalNAc-T2-GFP HeLa | -- | Human cervical cancer | DMEM + FBS (10%) + 1% Antibiotic cocktail | (Gift from Dr. Dibyendu Bhattacharya, ACTREC) |
| 4 | NIH/3T3 | CRL-1658™ | mouse embryonic fibroblasts | DMEM + BCS (10%) + 1% Antibiotic Cocktail | ATCC |

**List of cell-lines used**

- DMEM - Dulbecco's Modified Eagle's Medium (GIBCO By Life technologies Cat No.12800-17)
- RPMI 1640 - Roswell Park Memorial Institute Medium 1640 (GIBCO By Life technologies Cat No.23400-21)
- FBS- Fetal Bovine Serum (GIBCO By life technologies Cat No.26140-079)
- BCS- Bovine Calf Serum (Hyclone – SH30673.03)
- Antibiotic cocktail - (GIBCO By Life technologies Cat No.15240-062)

**Supplementary table 2:**

**List of 1004 deregulated genes along with annotations**

| Sr. No. | Gene | Description |
| --- | --- | --- |
| 1 | Gtdc2 | glycosyltransferase-like domain containing 2 |
| 2 | Col4a1 | collagen, type IV, alpha 1 |
| 3 | Aldh2 | aldehyde dehydrogenase 2, mitochondrial |
| 4 | Thumpd3 | THUMP domain containing 3 |
| 5 | Vmp1 | vacuole membrane protein 1 |
| 6 | Gorasp1 | golgi reassembly stacking protein 1 |
| 7 | Acox3 | acyl-Coenzyme A oxidase 3, pristanoyl |
| 8 | Fbxw26 | F-box and WD-40 domain protein 26 |
| 9 | Cpne8 | copine VIII |
| 10 | Shmt2 | serine hydroxymethyltransferase 2 (mitochondrial) |
| 11 | Plod3 | procollagen-lysine, 2-oxoglutarate 5-dioxygenase 3 |
| 12 | Rhbdd2 | rhomboid domain containing 2 |
| 13 | Zfp219 | zinc finger protein 219 |
| 14 | Clic4 | chloride intracellular channel 4 (mitochondrial) |
| 15 | Trps1 | trichorhinophalangeal syndrome I (human) |
| 16 | Magee1 | melanoma antigen, family E, 1 |
| 17 | Zfp503 | zinc finger protein 503 |
| 18 | Pbk | PDZ binding kinase |
| 19 | Fam3c | family with sequence similarity 3, member C |
| 20 | Ercc6l | excision repair cross-complementing rodent repair deficiency complementation group 6 like |
| 21 | Lrrc73 | leucine rich repeat containing 73 |
| 22 | Ssbp1 | single-stranded DNA binding protein 1 |
| 23 | Tmcc2 | transmembrane and coiled-coil domains 2 |
| 24 | Creg1 | cellular repressor of E1A-stimulated genes 1 |
| 25 | Tmem129 | transmembrane protein 129 |
| 26 | Ldlrap1 | low density lipoprotein receptor adaptor protein 1 |
| 27 | Sdc1 | syndecan 1 |
| 28 | Ccdc92 | coiled-coil domain containing 92 |
| 29 | Slc25a29 | solute carrier family 25 (mitochondrial carrier, palmitoylcarnitine transporter), member 29 |
| 30 | Angel1 | angel homolog 1 (Drosophila) |
| 31 | Tyk2 | tyrosine kinase 2 |
| 32 | Ptdss2 | phosphatidylserine synthase 2 |
| 33 | Olfr862 | olfactory receptor 862 |
| 34 | Ndufaf4 | NADH dehydrogenase (ubiquinone) 1 alpha subcomplex, assembly factor 4 |
| 35 | Carhsp1 | calcium regulated heat stable protein 1 |
| 36 | Tfrc | transferrin receptor |
| 37 | Soat1 | sterol O-acyltransferase 1 |
| 38 | Pvrl1 | poliovirus receptor-related 1 |
| 39 | Trpm4 | transient receptor potential cation channel, subfamily M, member 4 |
| 40 | Ppp1r35 | protein phosphatase 1, regulatory subunit 35 |
| 41 | Epha2 | Eph receptor A2 |
| 42 | Grina | glutamate receptor, ionotropic, N-methyl D-aspartate-associated protein 1 (glutamate binding) |
| 43 | Wfs1 | Wolfram syndrome 1 homolog (human) |
| 44 | Dpt | dermatopontin |
| 45 | Cenpq | centromere protein Q |
| 46 | Det1 | de-etiolated homolog 1 (Arabidopsis) |
| 47 | Napa | N-ethylmaleimide sensitive fusion protein attachment protein alpha |
| 48 | Ttll1 | tubulin tyrosine ligase-like 1 |
| 49 | Gbp2 | guanylate binding protein 2 |
| 50 | Ncs1 | neuronal calcium sensor 1 |
| 51 | Cdca3 | cell division cycle associated 3 |
| 52 | Rb1 | retinoblastoma 1 |
| 53 | Gcc1 | golgi coiled coil 1 |
| 54 | Tap1 | transporter 1, ATP-binding cassette, sub-family B (MDR/TAP) |
| 55 | Cd24a | CD24a antigen |
| 56 | Hist3h2a | histone cluster 3, H2a |
| 57 | Pitpna | phosphatidylinositol transfer protein, alpha |
| 58 | Kif5a | kinesin family member 5A |
| 59 | Trim36 | tripartite motif-containing 36 |
| 60 | Synm | synemin, intermediate filament protein |
| 61 | Mmp9 | matrix metallopeptidase 9 |
| 62 | Acta2 | actin, alpha 2, smooth muscle, aorta |
| 63 | Anapc4 | anaphase promoting complex subunit 4 |
| 64 | Hells | helicase, lymphoid specific |
| 65 | Lmnb1 | lamin B1 |
| 66 | Qpctl | glutaminyl-peptide cyclotransferase-like |
| 67 | Cxcr7 | chemokine (C-X-C motif) receptor 7 |
| 68 | Fos | FBJ osteosarcoma oncogene |
| 69 | Ppm1g | protein phosphatase 1G (formerly 2C), magnesium-dependent, gamma isoform |
| 70 | Map3k3 | mitogen-activated protein kinase kinase kinase 3 |
| 71 | Endod1 | endonuclease domain containing 1 |
| 72 | Cenpo | centromere protein O |
| 73 | Psmb8 | proteasome (prosome, macropain) subunit, beta type 8 (large multifunctional peptidase 7) |
| 74 | Ptx3 | pentraxin related gene |
| 75 | Stard10 | START domain containing 10 |
| 76 | Zbtb45 | zinc finger and BTB domain containing 45 |
| 77 | Slc26a11 | solute carrier family 26, member 11 |
| 78 | Trmt1l | tRNA methyltransferase 1 like |
| 79 | Rad21 | RAD21 homolog (S. pombe) |
| 80 | Kdsr | 3-ketodihydrosphingosine reductase |
| 81 | Slc2a4 | solute carrier family 2 (facilitated glucose transporter), member 4 |
| 82 | Dusp11 | dual specificity phosphatase 11 (RNA/RNP complex 1-interacting) |
| 83 | Sult1a1 | sulfotransferase family 1A, phenol-preferring, member 1 |
| 84 | 2310061J03Rik | RIKEN cDNA 2310061J03 gene |
| 85 | Nr2c1 | nuclear receptor subfamily 2, group C, member 1 |
| 86 | AU018091 | expressed sequence AU018091 |
| 87 | Hist1h2ab | histone cluster 1, H2ab |
| 88 | Pex11a | peroxisomal biogenesis factor 11 alpha |
| 89 | Tas1r1 | taste receptor, type 1, member 1 |
| 90 | Zfp322a | zinc finger protein 322A |
| 91 | Cep70 | centrosomal protein 70 |
| 92 | Chrd | chordin |
| 93 | Amacr | alpha-methylacyl-CoA racemase |
| 94 | Celf5 | CUGBP, Elav-like family member 5 |
| 95 | Plk3 | polo-like kinase 3 |
| 96 | Zfp566 | zinc finger protein 566 |
| 97 | Dusp9 | dual specificity phosphatase 9 |
| 98 | Dtwd1 | DTW domain containing 1 |
| 99 | Gpt | glutamic pyruvic transaminase, soluble |
| 100 | Pdgfra | platelet derived growth factor receptor, alpha polypeptide |
| 101 | 1700026D08Rik | RIKEN cDNA 1700026D08 gene |
| 102 | Gclc | glutamate-cysteine ligase, catalytic subunit |
| 103 | 2310045N01Rik | RIKEN cDNA 2310045N01 gene |
| 104 | Cdr2 | cerebellar degeneration-related 2 |
| 105 | H2-K1 | histocompatibility 2, K1, K region |
| 106 | Pik3cd | phosphatidylinositol 3-kinase catalytic delta polypeptide |
| 107 | Arrdc4 | arrestin domain containing 4 |
| 108 | Wdpcp | WD repeat containing planar cell polarity effector |
| 109 | Rad23b | RAD23b homolog (S. cerevisiae) |
| 110 | Acy1 | aminoacylase 1 |
| 111 | Nr4a1 | nuclear receptor subfamily 4, group A, member 1 |
| 112 | Tiam2 | T cell lymphoma invasion and metastasis 2 |
| 113 | Zbtb22 | zinc finger and BTB domain containing 22 |
| 114 | Mns1 | meiosis-specific nuclear structural protein 1 |
| 115 | Eln | elastin |
| 116 | Kif26b | kinesin family member 26B |
| 117 | Col4a2 | collagen, type IV, alpha 2 |
| 118 | Atg2a | autophagy related 2A |
| 119 | Fam229a | family with sequence similarity 229, member A |
| 120 | Apoe | apolipoprotein E |
| 121 | Stard4 | StAR-related lipid transfer (START) domain containing 4 |
| 122 | Slc25a12 | solute carrier family 25 (mitochondrial carrier, Aralar), member 12 |
| 123 | Gnl3 | guanine nucleotide binding protein-like 3 (nucleolar) |
| 124 | Rnf144a | ring finger protein 144A |
| 125 | 2310047K21Rik | RIKEN cDNA 2310047K21 gene |
| 126 | Setbp1 | SET binding protein 1 |
| 127 | Ccl8 | chemokine (C-C motif) ligand 8 |
| 128 | Pqlc3 | PQ loop repeat containing |
| 129 | Arl14ep | ADP-ribosylation factor-like 14 effector protein |
| 130 | Thsd1 | thrombospondin, type I, domain 1 |
| 131 | Zkscan14 | zinc finger with KRAB and SCAN domains 14 |
| 132 | Mslnl | mesothelin-like |
| 133 | Fscn1 | fascin homolog 1, actin bundling protein (Strongylocentrotus purpuratus) |
| 134 | Tm7sf2 | transmembrane 7 superfamily member 2 |
| 135 | Selrc1 | Sel1 repeat containing 1 |
| 136 | Nceh1 | neutral cholesterol ester hydrolase 1 |
| 137 | Bid | BH3 interacting domain death agonist |
| 138 | Dok3 | docking protein 3 |
| 139 | Smpd3 | sphingomyelin phosphodiesterase 3, neutral |
| 140 | Coro1b | coronin, actin binding protein 1B |
| 141 | 2700049A03Rik | RIKEN cDNA 2700049A03 gene |
| 142 | Crot | carnitine O-octanoyltransferase |
| 143 | Ccno | cyclin O |
| 144 | Cdc42ep3 | CDC42 effector protein (Rho GTPase binding) 3 |
| 145 | Gnb4 | guanine nucleotide binding protein (G protein), beta 4 |
| 146 | Slc25a47 | solute carrier family 25, member 47 |
| 147 | Hpx | hemopexin |
| 148 | Pigu | phosphatidylinositol glycan anchor biosynthesis, class U |
| 149 | Sema7a | sema domain, immunoglobulin domain (Ig), and GPI membrane anchor, (semaphorin) 7A |
| 150 | Cenpp | centromere protein P |
| 151 | Kbtbd11 | kelch repeat and BTB (POZ) domain containing 11 |
| 152 | Col20a1 | collagen, type XX, alpha 1 |
| 153 | Mapre1 | microtubule-associated protein, RP/EB family, member 1 |
| 154 | Rnf181 | ring finger protein 181 |
| 155 | Peo1 | progressive external ophthalmoplegia 1 (human) |
| 156 | Arl5c | ADP-ribosylation factor-like 5C |
| 157 | Ankrd1 | ankyrin repeat domain 1 (cardiac muscle) |
| 158 | Hist1h1c | histone cluster 1, H1c |
| 159 | Tspyl4 | TSPY-like 4 |
| 160 | Tns1 | tensin 1 |
| 161 | Sulf2 | sulfatase 2 |
| 162 | Nat8 | N-acetyltransferase 8 (GCN5-related, putative) |
| 163 | Gtpbp1 | GTP binding protein 1 |
| 164 | Pacs1 | phosphofurin acidic cluster sorting protein 1 |
| 165 | Hist1h1b | histone cluster 1, H1b |
| 166 | Crip2 | cysteine rich protein 2 |
| 167 | Cdkn1a | cyclin-dependent kinase inhibitor 1A (P21) |
| 168 | Dusp2 | dual specificity phosphatase 2 |
| 169 | Tgoln2 | trans-golgi network protein 2 |
| 170 | Ppp2r5d | protein phosphatase 2, regulatory subunit B (B56), delta isoform |
| 171 | Tmem190 | transmembrane protein 190 |
| 172 | Riok2 | RIO kinase 2 (yeast) |
| 173 | Plscr1 | phospholipid scramblase 1 |
| 174 | Dolk | dolichol kinase |
| 175 | Hist1h2ak | histone cluster 1, H2ak |
| 176 | Dzank1 | double zinc ribbon and ankyrin repeat domains 1 |
| 177 | Rab3d | RAB3D, member RAS oncogene family |
| 178 | Myo19 | myosin XIX |
| 179 | Gss | glutathione synthetase |
| 180 | BC022687 | cDNA sequence BC022687 |
| 181 | Zmynd8 | zinc finger, MYND-type containing 8 |
| 182 | Drg2 | developmentally regulated GTP binding protein 2 |
| 183 | Tnfrsf21 | tumor necrosis factor receptor superfamily, member 21 |
| 184 | Izumo4 | IZUMO family member 4 |
| 185 | Trp53inp1 | transformation related protein 53 inducible nuclear protein 1 |
| 186 | Usp20 | ubiquitin specific peptidase 20 |
| 187 | Dars2 | aspartyl-tRNA synthetase 2 (mitochondrial) |
| 188 | Frmd8 | FERM domain containing 8 |
| 189 | Pik3r5 | phosphoinositide-3-kinase, regulatory subunit 5, p101 |
| 190 | Vwce | von Willebrand factor C and EGF domains |
| 191 | Tsc22d4 | TSC22 domain family, member 4 |
| 192 | Trip13 | thyroid hormone receptor interactor 13 |
| 193 | Becn1 | beclin 1, autophagy related |
| 194 | Nckipsd | NCK interacting protein with SH3 domain |
| 195 | Zfp414 | zinc finger protein 414 |
| 196 | Commd2 | COMM domain containing 2 |
| 197 | Nup98 | nucleoporin 98 |
| 198 | Ska1 | spindle and kinetochore associated complex subunit 1 |
| 199 | Srsf3 | serine/arginine-rich splicing factor 3 |
| 200 | Rhod | ras homolog gene family, member D |
| 201 | Pdlim2 | PDZ and LIM domain 2 |
| 202 | Tmed8 | transmembrane emp24 domain containing 8 |
| 203 | Ahcy | S-adenosylhomocysteine hydrolase |
| 204 | Tcirg1 | T cell, immune regulator 1, ATPase, H+ transporting, lysosomal V0 protein A3 |
| 205 | Lepre1 | leprecan 1 |
| 206 | Hexim1 | hexamethylene bis-acetamide inducible 1 |
| 207 | Ifrd1 | interferon-related developmental regulator 1 |
| 208 | 1200014J11Rik | RIKEN cDNA 1200014J11 gene |
| 209 | Ttc26 | tetratricopeptide repeat domain 26 |
| 210 | Parp14 | poly (ADP-ribose) polymerase family, member 14 |
| 211 | Klhl26 | kelch-like 26 |
| 212 | Fermt3 | fermitin family homolog 3 (Drosophila) |
| 213 | Gstm5 | glutathione S-transferase, mu 5 |
| 214 | Mus81 | MUS81 endonuclease homolog (yeast) |
| 215 | Irgq | immunity-related GTPase family, Q |
| 216 | Msln | mesothelin |
| 217 | Ptplb | protein tyrosine phosphatase-like (proline instead of catalytic arginine), member b |
| 218 | Ppil4 | peptidylprolyl isomerase (cyclophilin)-like 4 |
| 219 | Col3a1 | collagen, type III, alpha 1 |
| 220 | Rasl11a | RAS-like, family 11, member A |
| 221 | Acsl1 | acyl-CoA synthetase long-chain family member 1 |
| 222 | Zfp637 | zinc finger protein 637 |
| 223 | Pola1 | polymerase (DNA directed), alpha 1 |
| 224 | Msrb3 | methionine sulfoxide reductase B3 |
| 225 | C230052I12Rik | RIKEN cDNA C230052I12 gene |
| 226 | Bub1 | budding uninhibited by benzimidazoles 1 homolog (S. cerevisiae) |
| 227 | Fbxl20 | F-box and leucine-rich repeat protein 20 |
| 228 | Lpcat3 | lysophosphatidylcholine acyltransferase 3 |
| 229 | Abcb1b | ATP-binding cassette, sub-family B (MDR/TAP), member 1B |
| 230 | Gtl3 | gene trap locus 3 |
| 231 | Zbtb8a | zinc finger and BTB domain containing 8a |
| 232 | Vcl | vinculin |
| 233 | Zfp955b | zinc finger protein 955B |
| 234 | 2010204K13Rik | RIKEN cDNA 2010204K13 gene |
| 235 | Gatsl3 | GATS protein-like 3 |
| 236 | Reep5 | receptor accessory protein 5 |
| 237 | Ecm1 | extracellular matrix protein 1 |
| 238 | Pten | phosphatase and tensin homolog |
| 239 | Gm5595 | predicted gene 5595 |
| 240 | Arhgef28 | Rho guanine nucleotide exchange factor (GEF) 28 |
| 241 | Slc3a1 | solute carrier family 3, member 1 |
| 242 | Slc38a1 | solute carrier family 38, member 1 |
| 243 | Ccnb2 | cyclin B2 |
| 244 | Siglecg | sialic acid binding Ig-like lectin G |
| 245 | Fam212b | family with sequence similarity 212, member B |
| 246 | Trim30a | tripartite motif-containing 30A |
| 247 | Nt5dc2 | 5'-nucleotidase domain containing 2 |
| 248 | Smndc1 | survival motor neuron domain containing 1 |
| 249 | Fndc5 | fibronectin type III domain containing 5 |
| 250 | Slc12a7 | solute carrier family 12, member 7 |
| 251 | Amhr2 | anti-Mullerian hormone type 2 receptor |
| 252 | Hoxd4 | homeobox D4 |
| 253 | Ccna2 | cyclin A2 |
| 254 | 1500012F01Rik | RIKEN cDNA 1500012F01 gene |
| 255 | Fgf10 | fibroblast growth factor 10 |
| 256 | Ccng1 | cyclin G1 |
| 257 | Arl4a | ADP-ribosylation factor-like 4A |
| 258 | Lynx1 | Ly6/neurotoxin 1 |
| 259 | Tnnc2 | troponin C2, fast |
| 260 | Slc38a2 | solute carrier family 38, member 2 |
| 261 | Nsl1 | NSL1, MIND kinetochore complex component, homolog (S. cerevisiae) |
| 262 | AW549877 | expressed sequence AW549877 |
| 263 | Bace1 | beta-site APP cleaving enzyme 1 |
| 264 | Bahd1 | bromo adjacent homology domain containing 1 |
| 265 | Akap12 | A kinase (PRKA) anchor protein (gravin) 12 |
| 266 | Pbx2 | pre B cell leukemia homeobox 2 |
| 267 | Csf3 | colony stimulating factor 3 (granulocyte) |
| 268 | Odc1 | ornithine decarboxylase, structural 1 |
| 269 | Paqr4 | progestin and adipoQ receptor family member IV |
| 270 | Kif20a | kinesin family member 20A |
| 271 | Hoxa7 | homeobox A7 |
| 272 | Aqp1 | aquaporin 1 |
| 273 | Edem1 | ER degradation enhancer, mannosidase alpha-like 1 |
| 274 | Dio2 | deiodinase, iodothyronine, type II |
| 275 | Itfg2 | integrin alpha FG-GAP repeat containing 2 |
| 276 | 2810007J24Rik | RIKEN cDNA 2810007J24 gene |
| 277 | Slc19a2 | solute carrier family 19 (thiamine transporter), member 2 |
| 278 | Maf | avian musculoaponeurotic fibrosarcoma (v-maf) AS42 oncogene homolog |
| 279 | Ubqln4 | ubiquilin 4 |
| 280 | Grem2 | gremlin 2 homolog, cysteine knot superfamily (Xenopus laevis) |
| 281 | Stard5 | StAR-related lipid transfer (START) domain containing 5 |
| 282 | Ethe1 | ethylmalonic encephalopathy 1 |
| 283 | Pdcd6ip | programmed cell death 6 interacting protein |
| 284 | Slc41a2 | solute carrier family 41, member 2 |
| 285 | Tmem205 | transmembrane protein 205 |
| 286 | Vac14 | Vac14 homolog (S. cerevisiae) |
| 287 | Ctsf | cathepsin F |
| 288 | 1700019L03Rik | RIKEN cDNA 1700019L03 gene |
| 289 | Runx1 | runt related transcription factor 1 |
| 290 | Fchsd1 | FCH and double SH3 domains 1 |
| 291 | Gdpd5 | glycerophosphodiester phosphodiesterase domain containing 5 |
| 292 | Zscan12 | zinc finger and SCAN domain containing 12 |
| 293 | L3hypdh | L-3-hydroxyproline dehydratase (trans-) |
| 294 | Cdk16 | cyclin-dependent kinase 16 |
| 295 | Trim16 | tripartite motif-containing 16 |
| 296 | Hnrnpd | heterogeneous nuclear ribonucleoprotein D |
| 297 | Glipr1 | GLI pathogenesis-related 1 (glioma) |
| 298 | Elmo2 | engulfment and cell motility 2 |
| 299 | Cdk2ap2 | CDK2-associated protein 2 |
| 300 | H2-T23 | histocompatibility 2, T region locus 23 |
| 301 | Prkg2 | protein kinase, cGMP-dependent, type II |
| 302 | Abcd4 | ATP-binding cassette, sub-family D (ALD), member 4 |
| 303 | Adar | adenosine deaminase, RNA-specific |
| 304 | Rrp9 | RRP9, small subunit (SSU) processome component, homolog (yeast) |
| 305 | Rnf135 | ring finger protein 135 |
| 306 | Rrp12 | ribosomal RNA processing 12 homolog (S. cerevisiae) |
| 307 | Dgat2 | diacylglycerol O-acyltransferase 2 |
| 308 | Ubash3b | ubiquitin associated and SH3 domain containing, B |
| 309 | Txnl4b | thioredoxin-like 4B |
| 310 | 4930444A02Rik | RIKEN cDNA 4930444A02 gene |
| 311 | Cndp2 | CNDP dipeptidase 2 (metallopeptidase M20 family) |
| 312 | Slc25a39 | solute carrier family 25, member 39 |
| 313 | Fcgrt | Fc receptor, IgG, alpha chain transporter |
| 314 | Rasl11b | RAS-like, family 11, member B |
| 315 | Tprg | transformation related protein 63 regulated |
| 316 | Dido1 | death inducer-obliterator 1 |
| 317 | 2310061I04Rik | RIKEN cDNA 2310061I04 gene |
| 318 | Ube2c | ubiquitin-conjugating enzyme E2C |
| 319 | Hrh3 | histamine receptor H3 |
| 320 | Defb41 | defensin beta 41 |
| 321 | Lrrc17 | leucine rich repeat containing 17 |
| 322 | D130020L05Rik | RIKEN cDNA D130020L05 gene |
| 323 | 1700006J14Rik | RIKEN cDNA 1700006J14 gene |
| 324 | Aoc3 | amine oxidase, copper containing 3 |
| 325 | Cyp2f2 | cytochrome P450, family 2, subfamily f, polypeptide 2 |
| 326 | Mki67 | antigen identified by monoclonal antibody Ki 67 |
| 327 | Srsf1 | serine/arginine-rich splicing factor 1 |
| 328 | Cables2 | CDK5 and Abl enzyme substrate 2 |
| 329 | Pkp1 | plakophilin 1 |
| 330 | Pex6 | peroxisomal biogenesis factor 6 |
| 331 | Exoc3l2 | exocyst complex component 3-like 2 |
| 332 | Pfkl | phosphofructokinase, liver, B-type |
| 333 | Acta1 | actin, alpha 1, skeletal muscle |
| 334 | 4930503L19Rik | RIKEN cDNA 4930503L19 gene |
| 335 | Aldh4a1 | aldehyde dehydrogenase 4 family, member A1 |
| 336 | Zcchc7 | zinc finger, CCHC domain containing 7 |
| 337 | Efhc1 | EF-hand domain (C-terminal) containing 1 |
| 338 | Wrap53 | WD repeat containing, antisense to TP53 |
| 339 | Ankrd42 | ankyrin repeat domain 42 |
| 340 | Egr4 | early growth response 4 |
| 341 | Grin3b | glutamate receptor, ionotropic, NMDA3B |
| 342 | Slc25a24 | solute carrier family 25 (mitochondrial carrier, phosphate carrier), member 24 |
| 343 | Thra | thyroid hormone receptor alpha |
| 344 | Ccl9 | chemokine (C-C motif) ligand 9 |
| 345 | Zfp81 | zinc finger protein 81 |
| 346 | Stx6 | syntaxin 6 |
| 347 | Nudt13 | nudix (nucleoside diphosphate linked moiety X)-type motif 13 |
| 348 | Ptprn | protein tyrosine phosphatase, receptor type, N |
| 349 | Leng1 | leukocyte receptor cluster (LRC) member 1 |
| 350 | Tmem180 | transmembrane protein 180 |
| 351 | Myc | myelocytomatosis oncogene |
| 352 | Tnfrsf11b | tumor necrosis factor receptor superfamily, member 11b (osteoprotegerin) |
| 353 | Pa2g4 | proliferation-associated 2G4 |
| 354 | Fam13a | family with sequence similarity 13, member A |
| 355 | Ccl11 | chemokine (C-C motif) ligand 11 |
| 356 | Dnajb2 | DnaJ (Hsp40) homolog, subfamily B, member 2 |
| 357 | Pcnt | pericentrin (kendrin) |
| 358 | U2af1 | U2 small nuclear ribonucleoprotein auxiliary factor (U2AF) 1 |
| 359 | Lpl | lipoprotein lipase |
| 360 | Amigo3 | adhesion molecule with Ig like domain 3 |
| 361 | Ric8 | resistance to inhibitors of cholinesterase 8 homolog (C. elegans) |
| 362 | Actr1b | ARP1 actin-related protein 1B, centractin beta |
| 363 | Vti1a | vesicle transport through interaction with t-SNAREs 1A |
| 364 | Emilin1 | elastin microfibril interfacer 1 |
| 365 | Atf1 | activating transcription factor 1 |
| 366 | Cebpd | CCAAT/enhancer binding protein (C/EBP), delta |
| 367 | Efnb1 | ephrin B1 |
| 368 | Rnf213 | ring finger protein 213 |
| 369 | Stip1 | stress-induced phosphoprotein 1 |
| 370 | Hsdl1 | hydroxysteroid dehydrogenase like 1 |
| 371 | Scara5 | scavenger receptor class A, member 5 (putative) |
| 372 | Serpinf1 | serine (or cysteine) peptidase inhibitor, clade F, member 1 |
| 373 | Bbc3 | BCL2 binding component 3 |
| 374 | Abcb4 | ATP-binding cassette, sub-family B (MDR/TAP), member 4 |
| 375 | Arfgap2 | ADP-ribosylation factor GTPase activating protein 2 |
| 376 | Fam133b | family with sequence similarity 133, member B |
| 377 | Hspa12b | heat shock protein 12B |
| 378 | Flt1 | FMS-like tyrosine kinase 1 |
| 379 | 1190002F15Rik | RIKEN cDNA 1190002F15 gene |
| 380 | Cercam | cerebral endothelial cell adhesion molecule |
| 381 | Mest | mesoderm specific transcript |
| 382 | Pms1 | postmeiotic segregation increased 1 (S. cerevisiae) |
| 383 | Sphk1 | sphingosine kinase 1 |
| 384 | Mt1 | metallothionein 1 |
| 385 | Tmem194 | transmembrane protein 194 |
| 386 | Angpt2 | angiopoietin 2 |
| 387 | Dpagt1 | dolichyl-phosphate (UDP-N-acetylglucosamine) acetylglucosaminephosphotransferase 1 (GlcNAc-1-P transferase) |
| 388 | Engase | endo-beta-N-acetylglucosaminidase |
| 389 | Larp4 | La ribonucleoprotein domain family, member 4 |
| 390 | Preb | prolactin regulatory element binding |
| 391 | Icam5 | intercellular adhesion molecule 5, telencephalin |
| 392 | Slc7a7 | solute carrier family 7 (cationic amino acid transporter, y+ system), member 7 |
| 393 | Pmaip1 | phorbol-12-myristate-13-acetate-induced protein 1 |
| 394 | Lrp10 | low-density lipoprotein receptor-related protein 10 |
| 395 | Cfb | complement factor B |
| 396 | Lamb2 | laminin, beta 2 |
| 397 | Sgol1 | shugoshin-like 1 (S. pombe) |
| 398 | Ufsp1 | UFM1-specific peptidase 1 |
| 399 | Hras1 | Harvey rat sarcoma virus oncogene 1 |
| 400 | Itpka | inositol 1,4,5-trisphosphate 3-kinase A |
| 401 | Pcdhb22 | protocadherin beta 22 |
| 402 | Gm5176 | high mobility group box 2 pseudogene |
| 403 | Grb7 | growth factor receptor bound protein 7 |
| 404 | Socs5 | suppressor of cytokine signaling 5 |
| 405 | B9d2 | B9 protein domain 2 |
| 406 | Sbk1 | SH3-binding kinase 1 |
| 407 | Tnrc6a | trinucleotide repeat containing 6a |
| 408 | Tmc7 | transmembrane channel-like gene family 7 |
| 409 | Igfbp4 | insulin-like growth factor binding protein 4 |
| 410 | Tgoln1 | trans-golgi network protein |
| 411 | Trp63 | transformation related protein 63 |
| 412 | BC055324 | cDNA sequence BC055324 |
| 413 | Procr | protein C receptor, endothelial |
| 414 | Asap1 | ArfGAP with SH3 domain, ankyrin repeat and PH domain1 |
| 415 | Rpp38 | ribonuclease P/MRP 38 subunit |
| 416 | Mtbp | Mdm2, transformed 3T3 cell double minute p53 binding protein |
| 417 | Dhx38 | DEAH (Asp-Glu-Ala-His) box polypeptide 38 |
| 418 | Zbtb12 | zinc finger and BTB domain containing 12 |
| 419 | Dpy30 | dpy-30 homolog (C. elegans) |
| 420 | Rftn2 | raftlin family member 2 |
| 421 | Pard3 | par-3 (partitioning defective 3) homolog (C. elegans) |
| 422 | S1pr1 | sphingosine-1-phosphate receptor 1 |
| 423 | Rab15 | RAB15, member RAS oncogene family |
| 424 | Smu1 | smu-1 suppressor of mec-8 and unc-52 homolog (C. elegans) |
| 425 | Rarres2 | retinoic acid receptor responder (tazarotene induced) 2 |
| 426 | Spatc1l | spermatogenesis and centriole associated 1 like |
| 427 | Amigo2 | adhesion molecule with Ig like domain 2 |
| 428 | Il15 | interleukin 15 |
| 429 | Rmdn3 | regulator of microtubule dynamics 3 |
| 430 | Crip3 | cysteine-rich protein 3 |
| 431 | Ces2g | carboxylesterase 2G |
| 432 | Zfp828 | zinc finger protein 828 |
| 433 | Soga3 | SOGA family member 3 |
| 434 | Cnep1r1 | CTD nuclear envelope phosphatase 1 regulatory subunit 1 |
| 435 | N4bp2l1 | NEDD4 binding protein 2-like 1 |
| 436 | Sesn3 | sestrin 3 |
| 437 | Hey1 | hairy/enhancer-of-split related with YRPW motif 1 |
| 438 | Fam198b | family with sequence similarity 198, member B |
| 439 | Zswim3 | zinc finger SWIM-type containing 3 |
| 440 | Cc2d2a | coiled-coil and C2 domain containing 2A |
| 441 | Mettl15 | methyltransferase like 15 |
| 442 | Tmcc3 | transmembrane and coiled coil domains 3 |
| 443 | Afmid | arylformamidase |
| 444 | Irgm1 | immunity-related GTPase family M member 1 |
| 445 | Ptdss1 | phosphatidylserine synthase 1 |
| 446 | Dnajc22 | DnaJ (Hsp40) homolog, subfamily C, member 22 |
| 447 | Cth | cystathionase (cystathionine gamma-lyase) |
| 448 | 1700003E16Rik | RIKEN cDNA 1700003E16 gene |
| 449 | Haus3 | HAUS augmin-like complex, subunit 3 |
| 450 | Parp2 | poly (ADP-ribose) polymerase family, member 2 |
| 451 | Slc41a1 | solute carrier family 41, member 1 |
| 452 | Hoxa2 | homeobox A2 |
| 453 | Taf1 | TAF1 RNA polymerase II, TATA box binding protein (TBP)-associated factor |
| 454 | Ada | adenosine deaminase |
| 455 | Steap4 | STEAP family member 4 |
| 456 | Fhl2 | four and a half LIM domains 2 |
| 457 | Zfp90 | zinc finger protein 90 |
| 458 | Gas6 | growth arrest specific 6 |
| 459 | Fut7 | fucosyltransferase 7 |
| 460 | Dynll1 | dynein light chain LC8-type 1 |
| 461 | Fbxw9 | F-box and WD-40 domain protein 9 |
| 462 | Tnfaip2 | tumor necrosis factor, alpha-induced protein 2 |
| 463 | Nrbp2 | nuclear receptor binding protein 2 |
| 464 | Npc2 | Niemann Pick type C2 |
| 465 | Gch1 | GTP cyclohydrolase 1 |
| 466 | Tsc22d1 | TSC22 domain family, member 1 |
| 467 | Mogat2 | monoacylglycerol O-acyltransferase 2 |
| 468 | Cd59a | CD59a antigen |
| 469 | Tmem50b | transmembrane protein 50B |
| 470 | Rbm4 | RNA binding motif protein 4 |
| 471 | Hat1 | histone aminotransferase 1 |
| 472 | Zfp292 | zinc finger protein 292 |
| 473 | Gm2a | GM2 ganglioside activator protein |
| 474 | Smarcad1 | SWI/SNF-related, matrix-associated actin-dependent regulator of chromatin, subfamily a, containing DEAD/H box 1 |
| 475 | Dlx2 | distal-less homeobox 2 |
| 476 | Trdmt1 | tRNA aspartic acid methyltransferase 1 |
| 477 | Ovgp1 | oviductal glycoprotein 1 |
| 478 | Stx11 | syntaxin 11 |
| 479 | Fbxw8 | F-box and WD-40 domain protein 8 |
| 480 | Tmem123 | transmembrane protein 123 |
| 481 | Ehd4 | EH-domain containing 4 |
| 482 | Arhgap12 | Rho GTPase activating protein 12 |
| 483 | Mertk | c-mer proto-oncogene tyrosine kinase |
| 484 | Nfkbie | nuclear factor of kappa light polypeptide gene enhancer in B cells inhibitor, epsilon |
| 485 | Ykt6 | YKT6 homolog (S. Cerevisiae) |
| 486 | 1190005I06Rik | RIKEN cDNA 1190005I06 gene |
| 487 | Col27a1 | collagen, type XXVII, alpha 1 |
| 488 | Tsen2 | tRNA splicing endonuclease 2 homolog (S. cerevisiae) |
| 489 | Rmnd5a | required for meiotic nuclear division 5 homolog A (S. cerevisiae) |
| 490 | Mipol1 | mirror-image polydactyly gene 1 homolog (human) |
| 491 | Naa16 | N(alpha)-acetyltransferase 16, NatA auxiliary subunit |
| 492 | Rdh14 | retinol dehydrogenase 14 (all-trans and 9-cis) |
| 493 | Nubp2 | nucleotide binding protein 2 |
| 494 | 2810474C18Rik | RIKEN cDNA 2810474C18 gene |
| 495 | Uspl1 | ubiquitin specific peptidase like 1 |
| 496 | Ptp4a1 | protein tyrosine phosphatase 4a1 |
| 497 | Mmrn2 | multimerin 2 |
| 498 | Ankrd9 | ankyrin repeat domain 9 |
| 499 | Anks3 | ankyrin repeat and sterile alpha motif domain containing 3 |
| 500 | Tmem141 | transmembrane protein 141 |
| 501 | Tmem115 | transmembrane protein 115 |
| 502 | Dclk1 | doublecortin-like kinase 1 |
| 503 | Ccl17 | chemokine (C-C motif) ligand 17 |
| 504 | Rusc1 | RUN and SH3 domain containing 1 |
| 505 | Pdxk-ps | pyridoxal (pyridoxine, vitamin B6) kinase, pseudogene |
| 506 | Map1lc3a | microtubule-associated protein 1 light chain 3 alpha |
| 507 | Rnd2 | Rho family GTPase 2 |
| 508 | Gstm2 | glutathione S-transferase, mu 2 |
| 509 | Rrm1 | ribonucleotide reductase M1 |
| 510 | Lrig3 | leucine-rich repeats and immunoglobulin-like domains 3 |
| 511 | Doc2g | double C2, gamma |
| 512 | Grk5 | G protein-coupled receptor kinase 5 |
| 513 | Cyp51 | cytochrome P450, family 51 |
| 514 | Dhx40 | DEAH (Asp-Glu-Ala-His) box polypeptide 40 |
| 515 | Tdp1 | tyrosyl-DNA phosphodiesterase 1 |
| 516 | Cdca2 | cell division cycle associated 2 |
| 517 | Mrvi1 | MRV integration site 1 |
| 518 | Gulp1 | GULP, engulfment adaptor PTB domain containing 1 |
| 519 | Fra10ac1 | FRA10AC1 homolog (human) |
| 520 | P2rx7 | purinergic receptor P2X, ligand-gated ion channel, 7 |
| 521 | Dok7 | docking protein 7 |
| 522 | Mgat4b | mannoside acetylglucosaminyltransferase 4, isoenzyme B |
| 523 | Glis2 | GLIS family zinc finger 2 |
| 524 | Ldb1 | LIM domain binding 1 |
| 525 | Glt8d2 | glycosyltransferase 8 domain containing 2 |
| 526 | Ltbp4 | latent transforming growth factor beta binding protein 4 |
| 527 | Fam131b | family with sequence similarity 131, member B |
| 528 | Snai1 | snail homolog 1 (Drosophila) |
| 529 | Nelfa | negative elongation factor complex member A, Whsc2 |
| 530 | E130012A19Rik | RIKEN cDNA E130012A19 gene |
| 531 | Hbegf | heparin-binding EGF-like growth factor |
| 532 | Necap2 | NECAP endocytosis associated 2 |
| 533 | Vip | vasoactive intestinal polypeptide |
| 534 | Tfap4 | transcription factor AP4 |
| 535 | Shpk | sedoheptulokinase |
| 536 | Rgcc | regulator of cell cycle |
| 537 | Mfsd2a | major facilitator superfamily domain containing 2A |
| 538 | Tsga14 | testis specific gene A14 |
| 539 | Tnfaip8l1 | tumor necrosis factor, alpha-induced protein 8-like 1 |
| 540 | Dstyk | dual serine/threonine and tyrosine protein kinase |
| 541 | Prkaa1 | protein kinase, AMP-activated, alpha 1 catalytic subunit |
| 542 | Retsat | retinol saturase (all trans retinol 13,14 reductase) |
| 543 | Zcchc8 | zinc finger, CCHC domain containing 8 |
| 544 | Bcl6 | B cell leukemia/lymphoma 6 |
| 545 | Smchd1 | SMC hinge domain containing 1 |
| 546 | Tgm2 | transglutaminase 2, C polypeptide |
| 547 | H2-Q2 | histocompatibility 2, Q region locus 2 |
| 548 | Foxc2 | forkhead box C2 |
| 549 | Noxred1 | NADP+ dependent oxidoreductase domain containing 1 |
| 550 | Actr8 | ARP8 actin-related protein 8 |
| 551 | Mgst1 | microsomal glutathione S-transferase 1 |
| 552 | Smcr7l | Smith-Magenis syndrome chromosome region, candidate 7-like (human) |
| 553 | 4933439C10Rik | RIKEN cDNA 4933439C10 gene |
| 554 | Nxph3 | neurexophilin 3 |
| 555 | Vegfc | vascular endothelial growth factor C |
| 556 | Zfp346 | zinc finger protein 346 |
| 557 | Dagla | diacylglycerol lipase, alpha |
| 558 | Brca2 | breast cancer 2 |
| 559 | Mcts2 | malignant T cell amplified sequence 2 |
| 560 | Fam192a | family with sequence similarity 192, member A |
| 561 | Cela1 | chymotrypsin-like elastase family, member 1 |
| 562 | Tagln | transgelin |
| 563 | Tcta | T cell leukemia translocation altered gene |
| 564 | Terf2ip | telomeric repeat binding factor 2, interacting protein |
| 565 | Zfp799 | zinc finger protein 799 |
| 566 | Klf1 | Kruppel-like factor 1 (erythroid) |
| 567 | Fam20c | family with sequence similarity 20, member C |
| 568 | Il2rb | interleukin 2 receptor, beta chain |
| 569 | Fam72a | family with sequence similarity 72, member A |
| 570 | Fam120b | family with sequence similarity 120, member B |
| 571 | Lpar2 | lysophosphatidic acid receptor 2 |
| 572 | Zeb2 | zinc finger E-box binding homeobox 2 |
| 573 | Abcb9 | ATP-binding cassette, sub-family B (MDR/TAP), member 9 |
| 574 | Arid5b | AT rich interactive domain 5B (MRF1-like) |
| 575 | Gp1bb | glycoprotein Ib, beta polypeptide |
| 576 | Atf3 | activating transcription factor 3 |
| 577 | Plcd4 | phospholipase C, delta 4 |
| 578 | Hsd17b1 | hydroxysteroid (17-beta) dehydrogenase 1 |
| 579 | Chd2 | chromodomain helicase DNA binding protein 2 |
| 580 | Stk16 | serine/threonine kinase 16 |
| 581 | Ccnl1 | cyclin L1 |
| 582 | Foxj1 | forkhead box J1 |
| 583 | Hyal1 | hyaluronoglucosaminidase 1 |
| 584 | Chi3l1 | chitinase 3-like 1 |
| 585 | Icam1 | intercellular adhesion molecule 1 |
| 586 | 8430408G22Rik | RIKEN cDNA 8430408G22 gene |
| 587 | Prkd2 | protein kinase D2 |
| 588 | Syncrip | synaptotagmin binding, cytoplasmic RNA interacting protein |
| 589 | Mob3a | MOB kinase activator 3A |
| 590 | Dffb | DNA fragmentation factor, beta subunit |
| 591 | Tab1 | TGF-beta activated kinase 1/MAP3K7 binding protein 1 |
| 592 | Gprc5a | G protein-coupled receptor, family C, group 5, member A |
| 593 | Mios | missing oocyte, meiosis regulator, homolog (Drosophila) |
| 594 | Evpl | envoplakin |
| 595 | Ccbe1 | collagen and calcium binding EGF domains 1 |
| 596 | Irf7 | interferon regulatory factor 7 |
| 597 | Nat6 | N-acetyltransferase 6 |
| 598 | Dok2 | docking protein 2 |
| 599 | Map2k6 | mitogen-activated protein kinase kinase 6 |
| 600 | Casp2 | caspase 2 |
| 601 | Cybasc3 | cytochrome b, ascorbate dependent 3 |
| 602 | Depdc1b | DEP domain containing 1B |
| 603 | Mtus2 | microtubule associated tumor suppressor candidate 2 |
| 604 | Nxpe4 | neurexophilin and PC-esterase domain family, member 4 |
| 605 | Zfp101 | zinc finger protein 101 |
| 606 | Klhl21 | kelch-like 21 |
| 607 | Wfdc18 | WAP four-disulfide core domain 18 |
| 608 | Fam57a | family with sequence similarity 57, member A |
| 609 | Atp13a2 | ATPase type 13A2 |
| 610 | Mfsd5 | major facilitator superfamily domain containing 5 |
| 611 | Rnf220 | ring finger protein 220 |
| 612 | Ostm1 | osteopetrosis associated transmembrane protein 1 |
| 613 | Ptges2 | prostaglandin E synthase 2 |
| 614 | Bgn | biglycan |
| 615 | Phyh | phytanoyl-CoA hydroxylase |
| 616 | Nucb2 | nucleobindin 2 |
| 617 | Foxn3 | forkhead box N3 |
| 618 | Iqcf1 | IQ motif containing F1 |
| 619 | Rnd1 | Rho family GTPase 1 |
| 620 | Clip1 | CAP-GLY domain containing linker protein 1 |
| 621 | Hnrpdl | heterogeneous nuclear ribonucleoprotein D-like |
| 622 | Utp23 | UTP23, small subunit (SSU) processome component, homolog (yeast) |
| 623 | Cotl1 | coactosin-like 1 (Dictyostelium) |
| 624 | Prkcd | protein kinase C, delta |
| 625 | 2810001G20Rik | RIKEN cDNA 2810001G20 gene |
| 626 | Pyroxd2 | pyridine nucleotide-disulphide oxidoreductase domain 2 |
| 627 | Mgp | matrix Gla protein |
| 628 | Chst7 | carbohydrate (N-acetylglucosamino) sulfotransferase 7 |
| 629 | Ttc32 | tetratricopeptide repeat domain 32 |
| 630 | Pkp2 | plakophilin 2 |
| 631 | Serpine2 | serine (or cysteine) peptidase inhibitor, clade E, member 2 |
| 632 | Klhdc8b | kelch domain containing 8B |
| 633 | Plod1 | procollagen-lysine, 2-oxoglutarate 5-dioxygenase 1 |
| 634 | Nckap5 | NCK-associated protein 5 |
| 635 | Osmr | oncostatin M receptor |
| 636 | 2010012O05Rik | RIKEN cDNA 2010012O05 gene |
| 637 | Nap1l2 | nucleosome assembly protein 1-like 2 |
| 638 | Tnik | TRAF2 and NCK interacting kinase |
| 639 | Mfsd11 | major facilitator superfamily domain containing 11 |
| 640 | Tmem64 | transmembrane protein 64 |
| 641 | Bmyc | brain expressed myelocytomatosis oncogene |
| 642 | Man1a | mannosidase 1, alpha |
| 643 | Sulf1 | sulfatase 1 |
| 644 | Entpd6 | ectonucleoside triphosphate diphosphohydrolase 6 |
| 645 | Zfyve16 | zinc finger, FYVE domain containing 16 |
| 646 | Rassf3 | Ras association (RalGDS/AF-6) domain family member 3 |
| 647 | Ifi27l2a | interferon, alpha-inducible protein 27 like 2A |
| 648 | Hist1h1e | histone cluster 1, H1e |
| 649 | Ltbp3 | latent transforming growth factor beta binding protein 3 |
| 650 | Tcp11l2 | t-complex 11 (mouse) like 2 |
| 651 | Phf17 | PHD finger protein 17 |
| 652 | Gpr176 | G protein-coupled receptor 176 |
| 653 | Dcxr | dicarbonyl L-xylulose reductase |
| 654 | Dnph1 | 2'-deoxynucleoside 5'-phosphate N-hydrolase 1 |
| 655 | Nudt22 | nudix (nucleoside diphosphate linked moiety X)-type motif 22 |
| 656 | Trmt1 | tRNA methyltransferase 1 |
| 657 | Ddx3y | DEAD (Asp-Glu-Ala-Asp) box polypeptide 3, Y-linked |
| 658 | Trip4 | thyroid hormone receptor interactor 4 |
| 659 | Cyp4f16 | cytochrome P450, family 4, subfamily f, polypeptide 16 |
| 660 | Srsf2 | serine/arginine-rich splicing factor 2 |
| 661 | Traip | TRAF-interacting protein |
| 662 | Lbx2 | ladybird homeobox homolog 2 (Drosophila) |
| 663 | Ptgfr | prostaglandin F receptor |
| 664 | Eva1a | eva-1 homolog A (C. elegans) |
| 665 | Kcne4 | potassium voltage-gated channel, Isk-related subfamily, gene 4 |
| 666 | C330018D20Rik | RIKEN cDNA C330018D20 gene |
| 667 | Cdc42bpb | CDC42 binding protein kinase beta |
| 668 | Asb4 | ankyrin repeat and SOCS box-containing 4 |
| 669 | Fdxr | ferredoxin reductase |
| 670 | Gorab | golgin, RAB6-interacting |
| 671 | Scaf8 | SR-related CTD-associated factor 8 |
| 672 | Nusap1 | nucleolar and spindle associated protein 1 |
| 673 | Fam222a | family with sequence similarity 222, member A |
| 674 | Calml4 | calmodulin-like 4 |
| 675 | Trib1 | tribbles homolog 1 (Drosophila) |
| 676 | Eftud2 | elongation factor Tu GTP binding domain containing 2 |
| 677 | Glrx5 | glutaredoxin 5 homolog (S. cerevisiae) |
| 678 | Atp2a2 | ATPase, Ca++ transporting, cardiac muscle, slow twitch 2 |
| 679 | Ccdc96 | coiled-coil domain containing 96 |
| 680 | Tpra1 | transmembrane protein, adipocyte asscociated 1 |
| 681 | Pex11c | peroxisomal biogenesis factor 11 gamma |
| 682 | Abcf2 | ATP-binding cassette, sub-family F (GCN20), member 2 |
| 683 | Rabggtb | RAB geranylgeranyl transferase, b subunit |
| 684 | Zfp472 | zinc finger protein 472 |
| 685 | Prl2c5 | prolactin family 2, subfamily c, member 5 |
| 686 | Tysnd1 | trypsin domain containing 1 |
| 687 | Tlcd2 | TLC domain containing 2 |
| 688 | Arhgef4 | Rho guanine nucleotide exchange factor (GEF) 4 |
| 689 | Dok4 | docking protein 4 |
| 690 | Prpf4b | PRP4 pre-mRNA processing factor 4 homolog B (yeast) |
| 691 | E130304I02Rik | RIKEN cDNA E130304I02 gene |
| 692 | Gas5 | growth arrest specific 5 |
| 693 | Islr | immunoglobulin superfamily containing leucine-rich repeat |
| 694 | Six5 | sine oculis-related homeobox 5 |
| 695 | Vcam1 | vascular cell adhesion molecule 1 |
| 696 | Crlf1 | cytokine receptor-like factor 1 |
| 697 | Rsph9 | radial spoke head 9 homolog (Chlamydomonas) |
| 698 | Ing5 | inhibitor of growth family, member 5 |
| 699 | Ikbip | IKBKB interacting protein |
| 700 | Foxred2 | FAD-dependent oxidoreductase domain containing 2 |
| 701 | Rchy1 | ring finger and CHY zinc finger domain containing 1 |
| 702 | Pinx1 | PIN2/TERF1 interacting, telomerase inhibitor 1 |
| 703 | Pds5b | PDS5, regulator of cohesion maintenance, homolog B (S. cerevisiae) |
| 704 | Adamts2 | a disintegrin-like and metallopeptidase (reprolysin type) with thrombospondin type 1 motif, 2 |
| 705 | Vash2 | vasohibin 2 |
| 706 | Plau | plasminogen activator, urokinase |
| 707 | Ptpn1 | protein tyrosine phosphatase, non-receptor type 1 |
| 708 | Fbxo5 | F-box protein 5 |
| 709 | Egln3 | EGL nine homolog 3 (C. elegans) |
| 710 | Supt3 | suppressor of Ty 3 |
| 711 | Atp6v0d1 | ATPase, H+ transporting, lysosomal V0 subunit D1 |
| 712 | Enpp3 | ectonucleotide pyrophosphatase/phosphodiesterase 3 |
| 713 | Pdrg1 | p53 and DNA damage regulated 1 |
| 714 | Serp2 | stress-associated endoplasmic reticulum protein family member 2 |
| 715 | Ripk3 | receptor-interacting serine-threonine kinase 3 |
| 716 | 4930506M07Rik | RIKEN cDNA 4930506M07 gene |
| 717 | Zfp521 | zinc finger protein 521 |
| 718 | Srrt | serrate RNA effector molecule homolog (Arabidopsis) |
| 719 | H2-Q1 | histocompatibility 2, Q region locus 1 |
| 720 | 1500026H17Rik | RIKEN cDNA 1500026H17 gene |
| 721 | Mxd1 | MAX dimerization protein 1 |
| 722 | Cpox | coproporphyrinogen oxidase |
| 723 | Ccdc18 | coiled-coil domain containing 18 |
| 724 | Man2b2 | mannosidase 2, alpha B2 |
| 725 | Desi1 | desumoylating isopeptidase 1 |
| 726 | Ckap2l | cytoskeleton associated protein 2-like |
| 727 | Cish | cytokine inducible SH2-containing protein |
| 728 | Phactr1 | phosphatase and actin regulator 1 |
| 729 | Hgfac | hepatocyte growth factor activator |
| 730 | Frat2 | frequently rearranged in advanced T cell lymphomas 2 |
| 731 | Arl6ip1 | ADP-ribosylation factor-like 6 interacting protein 1 |
| 732 | Cwf19l2 | CWF19-like 2, cell cycle control (S. pombe) |
| 733 | B230219D22Rik | RIKEN cDNA B230219D22 gene |
| 734 | H1f0 | H1 histone family, member 0 |
| 735 | Abhd16a | abhydrolase domain containing 16A |
| 736 | Nid2 | nidogen 2 |
| 737 | Sergef | secretion regulating guanine nucleotide exchange factor |
| 738 | Nanos1 | nanos homolog 1 (Drosophila) |
| 739 | Fmod | fibromodulin |
| 740 | Esyt1 | extended synaptotagmin-like protein 1 |
| 741 | Wfdc17 | WAP four-disulfide core domain 17 |
| 742 | Map3k6 | mitogen-activated protein kinase kinase kinase 6 |
| 743 | Zfp36l1 | zinc finger protein 36, C3H type-like 1 |
| 744 | Adamts1 | a disintegrin-like and metallopeptidase (reprolysin type) with thrombospondin type 1 motif, 1 |
| 745 | Gm10941 | predicted gene 10941 |
| 746 | Ifit2 | interferon-induced protein with tetratricopeptide repeats 2 |
| 747 | Chac1 | ChaC, cation transport regulator 1 |
| 748 | Pja2 | praja 2, RING-H2 motif containing |
| 749 | Cx3cl1 | chemokine (C-X3-C motif) ligand 1 |
| 750 | Socs1 | suppressor of cytokine signaling 1 |
| 751 | Cxcl1 | chemokine (C-X-C motif) ligand 1 |
| 752 | Jmjd7 | jumonji domain containing 7 |
| 753 | Parva | parvin, alpha |
| 754 | Slc35d2 | solute carrier family 35, member D2 |
| 755 | Rfxank | regulatory factor X-associated ankyrin-containing protein |
| 756 | Msl1 | male-specific lethal 1 homolog (Drosophila) |
| 757 | Snhg7 | small nucleolar RNA host gene (non-protein coding) 7 |
| 758 | Vrk3 | vaccinia related kinase 3 |
| 759 | 2210011C24Rik | RIKEN cDNA 2210011C24 gene |
| 760 | Opn3 | opsin 3 |
| 761 | Tuft1 | tuftelin 1 |
| 762 | Rras | Harvey rat sarcoma oncogene, subgroup R |
| 763 | Arrdc3 | arrestin domain containing 3 |
| 764 | Zbtb7c | zinc finger and BTB domain containing 7C |
| 765 | Lsm14b | LSM14 homolog B (SCD6, S. cerevisiae) |
| 766 | Sdc3 | syndecan 3 |
| 767 | Ikbke | inhibitor of kappaB kinase epsilon |
| 768 | Cyp2c55 | cytochrome P450, family 2, subfamily c, polypeptide 55 |
| 769 | Dnajb9 | DnaJ (Hsp40) homolog, subfamily B, member 9 |
| 770 | Ppbp | pro-platelet basic protein |
| 771 | B4galt1 | UDP-Gal:betaGlcNAc beta 1,4- galactosyltransferase, polypeptide 1 |
| 772 | Ddx17 | DEAD (Asp-Glu-Ala-Asp) box polypeptide 17 |
| 773 | Slc22a5 | solute carrier family 22 (organic cation transporter), member 5 |
| 774 | Whsc1 | Wolf-Hirschhorn syndrome candidate 1 (human) |
| 775 | Dlgap5 | discs, large (Drosophila) homolog-associated protein 5 |
| 776 | Tapbpl | TAP binding protein-like |
| 777 | Mrps18b | mitochondrial ribosomal protein S18B |
| 778 | Cytip | cytohesin 1 interacting protein |
| 779 | Ddx10 | DEAD (Asp-Glu-Ala-Asp) box polypeptide 10 |
| 780 | 4933436C20Rik | RIKEN cDNA 4933436C20 gene |
| 781 | Aif1l | allograft inflammatory factor 1-like |
| 782 | Nop58 | NOP58 ribonucleoprotein |
| 783 | Sdad1 | SDA1 domain containing 1 |
| 784 | Pygb | brain glycogen phosphorylase |
| 785 | Gls2 | glutaminase 2 (liver, mitochondrial) |
| 786 | Acot7 | acyl-CoA thioesterase 7 |
| 787 | Sfrp4 | secreted frizzled-related protein 4 |
| 788 | Nfkb2 | nuclear factor of kappa light polypeptide gene enhancer in B cells 2, p49/p100 |
| 789 | Slc1a5 | solute carrier family 1 (neutral amino acid transporter), member 5 |
| 790 | Pias3 | protein inhibitor of activated STAT 3 |
| 791 | Apitd1 | apoptosis-inducing, TAF9-like domain 1 |
| 792 | Nek2 | NIMA (never in mitosis gene a)-related expressed kinase 2 |
| 793 | Gltp | glycolipid transfer protein |
| 794 | Zscan29 | zinc finger SCAN domains 29 |
| 795 | Ndn | necdin |
| 796 | Agpat5 | 1-acylglycerol-3-phosphate O-acyltransferase 5 (lysophosphatidic acid acyltransferase, epsilon) |
| 797 | Has2 | hyaluronan synthase 2 |
| 798 | Stap2 | signal transducing adaptor family member 2 |
| 799 | Agtr2 | angiotensin II receptor, type 2 |
| 800 | Acy3 | aspartoacylase (aminoacylase) 3 |
| 801 | Plod2 | procollagen lysine, 2-oxoglutarate 5-dioxygenase 2 |
| 802 | Sparcl1 | SPARC-like 1 |
| 803 | Apoh | apolipoprotein H |
| 804 | Pcyox1 | prenylcysteine oxidase 1 |
| 805 | Cyp4f13 | cytochrome P450, family 4, subfamily f, polypeptide 13 |
| 806 | Slc25a25 | solute carrier family 25 (mitochondrial carrier, phosphate carrier), member 25 |
| 807 | Stom | stomatin |
| 808 | 2310047M10Rik | RIKEN cDNA 2310047M10 gene |
| 809 | Ppif | peptidylprolyl isomerase F (cyclophilin F) |
| 810 | Fbln1 | fibulin 1 |
| 811 | Vmac | vimentin-type intermediate filament associated coiled-coil protein |
| 812 | 8430427H17Rik | RIKEN cDNA 8430427H17 gene |
| 813 | Ipp | IAP promoted placental gene |
| 814 | Myl9 | myosin, light polypeptide 9, regulatory |
| 815 | Nupr1l | nuclear protein transcriptional regulator 1 like |
| 816 | Cdo1 | cysteine dioxygenase 1, cytosolic |
| 817 | Sec24d | Sec24 related gene family, member D (S. cerevisiae) |
| 818 | Diap2 | diaphanous homolog 2 (Drosophila) |
| 819 | Hic1 | hypermethylated in cancer 1 |
| 820 | C1rb | complement component 1, r subcomponent B |
| 821 | Zc3h12a | zinc finger CCCH type containing 12A |
| 822 | Seh1l | SEH1-like (S. cerevisiae |
| 823 | Mvp | major vault protein |
| 824 | Ddah2 | dimethylarginine dimethylaminohydrolase 2 |
| 825 | Peli2 | pellino 2 |
| 826 | Patl1 | protein associated with topoisomerase II homolog 1 (yeast) |
| 827 | Senp7 | SUMO1/sentrin specific peptidase 7 |
| 828 | Fam83d | family with sequence similarity 83, member D |
| 829 | Cdt1 | chromatin licensing and DNA replication factor 1 |
| 830 | Tmem186 | transmembrane protein 186 |
| 831 | Foxm1 | forkhead box M1 |
| 832 | Clcn2 | chloride channel 2 |
| 833 | Sap30 | sin3 associated polypeptide |
| 834 | Ftsj3 | FtsJ homolog 3 (E. coli) |
| 835 | Ifi35 | interferon-induced protein 35 |
| 836 | Cpb1 | carboxypeptidase B1 (tissue) |
| 837 | Mrpl38 | mitochondrial ribosomal protein L38 |
| 838 | Rbl2 | retinoblastoma-like 2 |
| 839 | Trim62 | tripartite motif-containing 62 |
| 840 | Insig2 | insulin induced gene 2 |
| 841 | Dcaf4 | DDB1 and CUL4 associated factor 4 |
| 842 | Ltb | lymphotoxin B |
| 843 | Snhg3 | small nucleolar RNA host gene (non-protein coding) 3 |
| 844 | Klf6 | Kruppel-like factor 6 |
| 845 | Cd93 | CD93 antigen |
| 846 | Wnk4 | WNK lysine deficient protein kinase 4 |
| 847 | Fam169b | family with sequence similarity 169, member B |
| 848 | Gmip | Gem-interacting protein |
| 849 | Pcdhb21 | protocadherin beta 21 |
| 850 | Cttn | cortactin |
| 851 | Neat1 | nuclear paraspeckle assembly transcript 1 (non-protein coding) |
| 852 | Dennd2c | DENN/MADD domain containing 2C |
| 853 | Atg12 | autophagy related 12 |
| 854 | Psmb9 | proteasome (prosome, macropain) subunit, beta type 9 (large multifunctional peptidase 2) |
| 855 | Actn3 | actinin alpha 3 |
| 856 | Grpr | gastrin releasing peptide receptor |
| 857 | Ttc30a1 | tetratricopeptide repeat domain 30A1 |
| 858 | Ddx25 | DEAD (Asp-Glu-Ala-Asp) box polypeptide 25 |
| 859 | Fam13c | family with sequence similarity 13, member C |
| 860 | Shisa4 | shisa homolog 4 (Xenopus laevis) |
| 861 | Jag1 | jagged 1 |
| 862 | Aldh6a1 | aldehyde dehydrogenase family 6, subfamily A1 |
| 863 | Arhgap18 | Rho GTPase activating protein 18 |
| 864 | Zfp763 | zinc finger protein 763 |
| 865 | Csrnp2 | cysteine-serine-rich nuclear protein 2 |
| 866 | Pgm5 | phosphoglucomutase 5 |
| 867 | Gas1 | growth arrest specific 1 |
| 868 | Wisp2 | WNT1 inducible signaling pathway protein 2 |
| 869 | Slc9a3r1 | solute carrier family 9 (sodium/hydrogen exchanger), member 3 regulator 1 |
| 870 | Itm2c | integral membrane protein 2C |
| 871 | Agap3 | ArfGAP with GTPase domain, ankyrin repeat and PH domain 3 |
| 872 | Vpreb1 | pre-B lymphocyte gene 1 |
| 873 | Ifi44 | interferon-induced protein 44 |
| 874 | Slc39a4 | solute carrier family 39 (zinc transporter), member 4 |
| 875 | Cxcl12 | chemokine (C-X-C motif) ligand 12 |
| 876 | Msantd1 | Myb/SANT-like DNA-binding domain containing 1 |
| 877 | Lpo | lactoperoxidase |
| 878 | B4galnt1 | beta-1,4-N-acetyl-galactosaminyl transferase 1 |
| 879 | Osgin2 | oxidative stress induced growth inhibitor family member 2 |
| 880 | Ppargc1a | peroxisome proliferative activated receptor, gamma, coactivator 1 alpha |
| 881 | Mt2 | metallothionein 2 |
| 882 | Fosl2 | fos-like antigen 2 |
| 883 | Hip1r | huntingtin interacting protein 1 related |
| 884 | Rad51b | RAD51 homolog B |
| 885 | Rep15 | RAB15 effector protein |
| 886 | Pold1 | polymerase (DNA directed), delta 1, catalytic subunit |
| 887 | Clec3b | C-type lectin domain family 3, member b |
| 888 | Spn | sialophorin |
| 889 | Dalrd3 | DALR anticodon binding domain containing 3 |
| 890 | Ogn | osteoglycin |
| 891 | Dcbld1 | discoidin, CUB and LCCL domain containing 1 |
| 892 | Kif18a | kinesin family member 18A |
| 893 | Papd4 | PAP associated domain containing 4 |
| 894 | Scn1b | sodium channel, voltage-gated, type I, beta |
| 895 | Atp6ap1 | ATPase, H+ transporting, lysosomal accessory protein 1 |
| 896 | Efnb3 | ephrin B3 |
| 897 | Gen1 | Gen homolog 1, endonuclease (Drosophila) |
| 898 | Prpf38b | PRP38 pre-mRNA processing factor 38 (yeast) domain containing B |
| 899 | Fyco1 | FYVE and coiled-coil domain containing 1 |
| 900 | Zfp410 | zinc finger protein 410 |
| 901 | Zfp276 | zinc finger protein (C2H2 type) 276 |
| 902 | Apol7a | apolipoprotein L 7a |
| 903 | Rdh10 | retinol dehydrogenase 10 (all-trans) |
| 904 | Ccdc84 | coiled-coil domain containing 84 |
| 905 | Copz2 | coatomer protein complex, subunit zeta 2 |
| 906 | Shd | src homology 2 domain-containing transforming protein D |
| 907 | Clk3 | CDC-like kinase 3 |
| 908 | 1600002K03Rik | RIKEN cDNA 1600002K03 gene |
| 909 | Rcan1 | regulator of calcineurin 1 |
| 910 | Il1r1 | interleukin 1 receptor, type I |
| 911 | Ints7 | integrator complex subunit 7 |
| 912 | Il1rl1 | interleukin 1 receptor-like 1 |
| 913 | Tex40 | testis expressed 40 |
| 914 | Pdxp | pyridoxal (pyridoxine, vitamin B6) phosphatase |
| 915 | Ampd2 | adenosine monophosphate deaminase 2 |
| 916 | Ltb4r1 | leukotriene B4 receptor 1 |
| 917 | Man2b1 | mannosidase 2, alpha B1 |
| 918 | Sirt5 | sirtuin 5 (silent mating type information regulation 2 homolog) 5 (S. cerevisiae) |
| 919 | Zfp329 | zinc finger protein 329 |
| 920 | Tbccd1 | TBCC domain containing 1 |
| 921 | Tmem38a | transmembrane protein 38A |
| 922 | Mlst8 | MTOR associated protein, LST8 homolog (S. cerevisiae) |
| 923 | Tmem223 | transmembrane protein 223 |
| 924 | Pomgnt1 | protein O-linked mannose beta1,2-N-acetylglucosaminyltransferase |
| 925 | Cdk12 | cyclin-dependent kinase 12 |
| 926 | Cdkn3 | cyclin-dependent kinase inhibitor 3 |
| 927 | Ebag9 | estrogen receptor-binding fragment-associated gene 9 |
| 928 | 6030419C18Rik | RIKEN cDNA 6030419C18 gene |
| 929 | Runx1t1 | runt-related transcription factor 1; translocated to, 1 (cyclin D-related) |
| 930 | Fzd4 | frizzled homolog 4 (Drosophila) |
| 931 | Cspg4 | chondroitin sulfate proteoglycan 4 |
| 932 | Snai2 | snail homolog 2 (Drosophila) |
| 933 | Dexi | dexamethasone-induced transcript |
| 934 | Ero1l | ERO1-like (S. cerevisiae) |
| 935 | Rhobtb3 | Rho-related BTB domain containing 3 |
| 936 | Saa1 | serum amyloid A 1 |
| 937 | Cdc73 | cell division cycle 73, Paf1/RNA polymerase II complex component |
| 938 | Edil3 | EGF-like repeats and discoidin I-like domains 3 |
| 939 | Mgst2 | microsomal glutathione S-transferase 2 |
| 940 | Rhbdl3 | rhomboid, veinlet-like 3 (Drosophila) |
| 941 | Depdc7 | DEP domain containing 7 |
| 942 | Tpm2 | tropomyosin 2, beta |
| 943 | Hn1 | hematological and neurological expressed sequence 1 |
| 944 | Nomo1 | nodal modulator 1 |
| 945 | Tamm41 | TAM41, mitochondrial translocator assembly and maintenance protein, homolog (S. cerevisiae) |
| 946 | Strip2 | striatin interacting protein 2 |
| 947 | Speg | SPEG complex locus |
| 948 | Fasn | fatty acid synthase |
| 949 | Itga8 | integrin alpha 8 |
| 950 | Zfp68 | zinc finger protein 68 |
| 951 | Sfrp2 | secreted frizzled-related protein 2 |
| 952 | Kif4 | kinesin family member 4 |
| 953 | 4930515G01Rik | RIKEN cDNA 4930515G01 gene |
| 954 | Mxra7 | matrix-remodelling associated 7 |
| 955 | Slc30a1 | solute carrier family 30 (zinc transporter), member 1 |
| 956 | Purg | purine-rich element binding protein G |
| 957 | Gna11 | guanine nucleotide binding protein, alpha 11 |
| 958 | Adat2 | adenosine deaminase, tRNA-specific 2 |
| 959 | Mat2a | methionine adenosyltransferase II, alpha |
| 960 | Nrf1 | nuclear respiratory factor 1 |
| 961 | Fam212a | family with sequence similarity 212, member A |
| 962 | Fbrsl1 | fibrosin-like 1 |
| 963 | Gm7265 | predicted gene 7265 |
| 964 | Btg2 | B cell translocation gene 2, anti-proliferative |
| 965 | Nr1i3 | nuclear receptor subfamily 1, group I, member 3 |
| 966 | Pogz | pogo transposable element with ZNF domain |
| 967 | Rbp7 | retinol binding protein 7, cellular |
| 968 | Fbxo28 | F-box protein 28 |
| 969 | Ttl | tubulin tyrosine ligase |
| 970 | Atpaf2 | ATP synthase mitochondrial F1 complex assembly factor 2 |
| 971 | Angptl2 | angiopoietin-like 2 |
| 972 | Tbl1xr1 | transducin (beta)-like 1X-linked receptor 1 |
| 973 | Wdr4 | WD repeat domain 4 |
| 974 | Oip5 | Opa interacting protein 5 |
| 975 | Ifi30 | interferon gamma inducible protein 30 |
| 976 | Aqp3 | aquaporin 3 |
| 977 | Homez | homeodomain leucine zipper-encoding gene |
| 978 | Nup133 | nucleoporin 133 |
| 979 | Trib2 | tribbles homolog 2 (Drosophila) |
| 980 | Tesk1 | testis specific protein kinase 1 |
| 981 | Smim3 | small integral membrane protein 3 |
| 982 | Peli3 | pellino 3 |
| 983 | Slc35e1 | solute carrier family 35, member E1 |
| 984 | Dusp10 | dual specificity phosphatase 10 |
| 985 | Tmem176a | transmembrane protein 176A |
| 986 | Alox5ap | arachidonate 5-lipoxygenase activating protein |
| 987 | Wipi2 | WD repeat domain, phosphoinositide interacting 2 |
| 988 | Abcc4 | ATP-binding cassette, sub-family C (CFTR/MRP), member 4 |
| 989 | Itgav | integrin alpha V |
| 990 | Snx21 | sorting nexin family member 21 |
| 991 | Tmem127 | transmembrane protein 127 |
| 992 | Fam5c | family with sequence similarity 5, member C |
| 993 | Ogg1 | 8-oxoguanine DNA-glycosylase 1 |
| 994 | Fign | fidgetin |
| 995 | Nr1d2 | nuclear receptor subfamily 1, group D, member 2 |
| 996 | Gm166 | predicted gene 166 |
| 997 | Armc7 | armadillo repeat containing 7 |
| 998 | Btbd9 | BTB (POZ) domain containing 9 |
| 999 | Apobr | apolipoprotein B receptor |
| 1000 | Stk25 | serine/threonine kinase 25 (yeast) |
| 1001 | Ctla2b | cytotoxic T lymphocyte-associated protein 2 beta |
| 1002 | Sc4mol | sterol-C4-methyl oxidase-like |
| 1003 | Gpaa1 | GPI anchor attachment protein 1 |
| 1004 | Aspn | asporin |

**Supplementary table 3:**

**Pathway analysis of 1004 deregulated genes.**

| Phagocytosis related pathways | ABC TRANSPORTERS | ABCD4, ABCB9, TAP1, ABCC4, ABCB4 |
| --- | --- | --- |
|  | ENDOCYTOSIS | PDGFRA, HRAS, FLT1, IL2RB, PARD3, GRK5, HLA-E, TFRC, ASAP1, PDCD6IP, LDLRAP1, EHD4, ARFGAP2 |
|  | LYSOSOME | ABCB9, MAN2B1, TCIRG1, ATP6AP1, ATP6V0D1, HYAL1, NPC2, GM2A, CTSF |
|  | PEROXISOME | ABCD4, ACOX3, ACSL1, AMACR, PEX6, PHYH, CROT, PEX11A |
|  | SNARE INTERACTIONS IN VESICULAR TRANSPORT | STX6, YKT6, VTI1A, STX11 |
| Cell cycle and DNA damage related pathways | CELL_CYCLE | NUP133, SEH1L, PPP2R5D, KIF18A, PSMB8, KIF20A, PSMB9, CDKN1A, DYNLL1, UBE2C, ANAPC4, CENPP, CENPQ, CENPO, RAD21, MAPRE1, SGOL1, SKA1, NSL1, APITD1, ERCC6L, CLIP1, BUB1, B9D2, POLA1, CDT1, FBXO5, GORASP1, POLD1, CCNA2, RB1, NEK2, PCNT, CEP70, CEP41, CCNB2, MYC, RBL2, LMNB1, HIST1H2AB, OIP5, TERF2IP, DIDO1, WRAP53 |
|  | CELL_CYCLE_MITOTIC | NUP133, SEH1L, PPP2R5D, KIF18A, PSMB8, KIF20A, PSMB9, CDKN1A, DYNLL1, UBE2C, ANAPC4, CENPP, CENPQ, CENPO, RAD21, MAPRE1, SGOL1, SKA1, NSL1, APITD1, ERCC6L, CLIP1, BUB1, B9D2, POLA1, CDT1, FBXO5, GORASP1, POLD1, CCNA2, RB1, NEK2, PCNT, CEP70, CEP41, CCNB2, MYC, RBL2 |
|  | DNA_REPLICATION | NUP133, SEH1L, PPP2R5D, KIF18A, PSMB8, KIF20A, PSMB9, CDKN1A, CENPP, CENPQ, CENPO, RAD21, MAPRE1, SGOL1, SKA1, NSL1, APITD1, ERCC6L, CLIP1, BUB1, B9D2, POLA1, CDT1, FBXO5, GORASP1, POLD1, CCNA2, RB1 |
|  | MITOTIC_M_M_G1_PHASES | NUP133, SEH1L, PPP2R5D, KIF18A, PSMB8, KIF20A, PSMB9, CENPP, CENPQ, CENPO, RAD21, MAPRE1, SGOL1, SKA1, NSL1, APITD1, ERCC6L, CLIP1, BUB1, B9D2, POLA1, CDT1, FBXO5, GORASP1 |
|  | MITOTIC_PROMETAPHASE | NUP133, SEH1L, PPP2R5D, KIF18A, CENPP, CENPQ, CENPO, RAD21, MAPRE1, SGOL1, SKA1, NSL1, APITD1, ERCC6L, CLIP1, BUB1, B9D2 |
|  | APOPTOSIS | PSMB8, PSMB9, DYNLL1, LMNB1, PRKCD, DFFB, H1F0, HIST1H1C, HIST1H1E, HIST1H1B, PKP1, BBC3, PMAIP1, BID |
|  | APOPTOSIS_INDUCED_DNA_FRAGMENTATION | DFFB, H1F0, HIST1H1C, HIST1H1E, HIST1H1B |
|  | CHROMOSOME_MAINTENANCE | CENPP, CENPQ, CENPO, RAD21, POLA1, POLD1, LMNB1, HIST1H2AB, OIP5, TERF2IP, DIDO1, WRAP53 |
|  | APOPTOTIC_EXECUTION_PHASE | LMNB1, PRKCD, DFFB, H1F0, HIST1H1C, HIST1H1E, HIST1H1B, PKP1 |
|  | MITOTIC_G2_G2_M_PHASES | DYNLL1, MAPRE1, CCNA2, NEK2, PCNT, CEP70, CEP41, CCNB2 |
| Inflammation related pathways | IMMUNE_SYSTEM | NUP133, SEH1L, PPP2R5D, KIF18A, PSMB8, KIF20A, PSMB9, CDKN1A, DYNLL1, UBE2C, ANAPC4, PIK3CD, PTEN, HRAS, PRKCD, IRF7, PTPN1, SOCS1, ICAM1, VCAM1, TYK2, GBP2, ADAR, IFI35, IFIT2, MT2A, MAP2K6, TAB1, NFKB2, PELI3, PELI2, IL1R1, MAP3K3, IL2RB, CISH, ITGAV, KIF4A, KIF5A, NR4A1, MLST8, SEC24D, RCHY1, FBXW8, ASB4, DET1, RNF220, TRIM36, PJA2, TAP1, ACTR1B, CTSF, NFKBIE, SAA1, ATF1, FOS, IKBKE, RIPK3, RNF135, ATG12, CASP2, P2RX7, DEFB130, CFB |
|  | CYTOKINE_SIGNALING_IN_IMMUNE_SYSTEM | NUP133, SEH1L, PSMB8, PIK3CD, HRAS, PRKCD, IRF7, PTPN1, SOCS1, ICAM1, VCAM1, TYK2, GBP2, ADAR, IFI35, IFIT2, MT2A, MAP2K6, TAB1, NFKB2, PELI3, PELI2, IL1R1, MAP3K3, IL2RB, CISH |
|  | ADAPTIVE_IMMUNE_SYSTEM | PPP2R5D, KIF18A, PSMB8, KIF20A, PSMB9, CDKN1A, DYNLL1, UBE2C, ANAPC4, PIK3CD, PTEN, HRAS, SOCS1, ICAM1, VCAM1, ITGAV, KIF4A, KIF5A, NR4A1, MLST8, SEC24D, RCHY1, FBXW8, ASB4, DET1, RNF220, TRIM36, PJA2, TAP1, ACTR1B, CTSF, NFKBIE |
|  | INTERFERON_SIGNALING | NUP133, SEH1L, PSMB8, PRKCD, IRF7, PTPN1, SOCS1, ICAM1, VCAM1, TYK2, GBP2, ADAR, IFI35, IFIT2, MT2A |
|  | TRIF_MEDIATED_TLR3_SIGNALING | PPP2R5D, IRF7, MAP2K6, TAB1, NFKB2, SAA1, ATF1, FOS, IKBKE, RIPK3 |
|  | ACTIVATED_TLR4_SIGNALLING | PPP2R5D, IRF7, MAP2K6, TAB1, NFKB2, PELI3, PELI2, SAA1, ATF1, FOS, IKBKE |
|  | INTERFERON_ALPHA_BETA_SIGNALING | PSMB8, IRF7, PTPN1, SOCS1, TYK2, GBP2, ADAR, IFI35, IFIT2 |
|  | SIGNALING_BY_ILS | PIK3CD, HRAS, TYK2, MAP2K6, TAB1, NFKB2, PELI3, PELI2, IL1R1, MAP3K3, IL2RB |
|  | INNATE_IMMUNE_SYSTEM | PPP2R5D, IRF7, MAP2K6, TAB1, NFKB2, PELI3, PELI2, SAA1, ATF1, FOS, IKBKE, RIPK3, RNF135, ATG12, CASP2, P2RX7, DEFB130, CFB |
|  | TRAF6_MEDIATED_INDUCTION_OF_NFKB_AND_MAP_KINASES_UPON_TLR7_8_OR_9_ACTIVATION | PPP2R5D, MAP2K6, TAB1, NFKB2, PELI3, PELI2, SAA1, ATF1, FOS |
|  | TOLL_RECEPTOR_CASCADES | PPP2R5D, IRF7, MAP2K6, TAB1, NFKB2, PELI3, PELI2, SAA1, ATF1, FOS, IKBKE |
|  | INTERFERON_GAMMA_SIGNALING | PRKCD, IRF7, PTPN1, SOCS1, ICAM1, VCAM1, GBP2, MT2A |
|  | IL1_SIGNALING | MAP2K6, TAB1, PELI3, PELI2, IL1R1, MAP3K3 |
|  | CHEMOKINE_RECEPTORS_BIND_CHEMOKINES | PPBP, CXCL12, CXCL1, CXCR7, CCL11, CCL17, CX3CL1 |
|  | CLASS_I_MHC_MEDIATED_ANTIGEN_PROCESSING_PRESENTATION | PSMB8, PSMB9, UBE2C, ANAPC4, SOCS1, ITGAV, SEC24D, RCHY1, FBXW8, ASB4, DET1, RNF220, TRIM36, PJA2, TAP1 |
|  | INTEGRIN_CELL_SURFACE_INTERACTIONS | PTPN1, ICAM1, VCAM1, ITGAV, COL4A1, COL4A2, LAMB2, ITGA8 |

**Supplementary table 4:**

**Tabulation of human *Alu* elements detected computationally from clones E7 and B10 using next generation sequencing.**

|  | **Alu-J** | **Alu-Sx** | **Alu-Sc** | **Alu-Sb** | **Alu-Sq** | **Alu-Sp** | **Alu-FLA?** | **Alu-Spqx** | **Total** |
| --- | --- | --- | --- | --- | --- | --- | --- | --- | --- |
| **E7** | 33 | 27 | 22 | 13 | 13 | 9 | 2 | 2 | 121 |
| **B10** | 36 | 21 | 15 | 9 | 3 | 3 | 1 |  | 88 |
| **Total** | 69 | 48 | 37 | 22 | 16 | 12 | 3 | 2 | 209 |

Human *Alu* elements specific to clones E7 and B10 were identified by depleting the genebank accession ids of human *Alu* sequences found in the control (SRA ERP000354) reads. NGS reads representing more than 80% identity to human *Alu* elements were taken as putative human *Alu* elements.

**Supplementary table 5:**

**List of antibodies used**

| Sr. No | Antibody | Source | Catalogue No. |
| --- | --- | --- | --- |
| 1 | NF-κB | Abcam®, UK. | ab16502 |
| 2 | IL-6 | Abcam®, UK. | ab6672 |
| 3 | TNF-α | ThermoFischer Scientific,USA. | PA5-19810 |
| 4 | IFN-γ | ThermoFischer Scientific, USA. | PA1-24782 |
| 5 | FITC-labeled anti-rabbit secondary antibody | Abcam®, UK. | ab6717 |
| 6 | FITC-labeled anti-mouse secondary antibody | Abcam®, UK. | ab6785 |
| 7 | FITC-labeled anti-goat secondary antibody | Abcam®, UK. | ab7121 |
| 8 | TRITC | Abcam® UK. | Ab6883 |
| 9 | Rhodamine | Merck Millipore, USA. | AP160R |
